# Green synthesis, characterization, anti-SARS-CoV-2 entry, and replication of lactoferrin-coated zinc nanoparticles with halting lung fibrosis induced in adult male albino rats

**DOI:** 10.1038/s41598-023-42702-0

**Published:** 2023-09-23

**Authors:** Esmail M. El-Fakharany, Yousra A. El-Maradny, Mahmoud Ashry, Khaled G. Abdel-Wahhab, Marwa E. Shabana, Hamada El-Gendi

**Affiliations:** 1 Protein Research Department, Genetic Engineering and Biotechnology Research Institute (GEBRI), City of Scientific Research and Technological Applications (SRTA-City), New Borg El-Arab City, Alexandria 21934 Egypt; 2 Microbiology and Immunology, Faculty of Pharmacy, Arab Academy for Science, Technology and Maritime Transport (AASTMT), Alamein, 51718 Egypt; 3 Zoology Department, Faculty of Science, Al-Azhar University, Assuit, Egypt; 4 Medical Physiology Department, National Research Centre, Giza, Egypt; 5 Pathology Department, National Research Centre, Giza, Egypt; 6 Bioprocess Development Department, Genetic Engineering and Biotechnology Research Institute, City of Scientific Research and Technological Applications (SRTA-City), New Borg El-Arab City, Alexandria 21934 Egypt

**Keywords:** Biochemistry, Biotechnology, Drug discovery, Microbiology

## Abstract

The ethanolic extract of *Coleus forskohlii* Briq leaves was employed in the green synthesis of zinc nanoparticles (Zn-NPs) by an immediate, one-step, and cost-effective method in the present study. Zn-NPs were coated with purified bovine lactoferrin (LF) and characterized through different instrumental analysis. The biosynthesized Zn-NPs were white in color revealing oval to spherical-shaped particles with an average size of 77 ± 5.50 nm, whereas LF-coated Zn-NPs (LF-Zn-NPs) revealed a larger particles size of up to 98 ± 6.40 nm. The biosynthesized Zn-NPs and LF-Zn-NPs revealed negatively charged surfaces with zeta-potentials of – 20.25 ± 0.35 and – 44.3 ± 3.25 mV, respectively. Interestingly, the LF-Zn-NPs showed potent in vitro retardation for SARS-CoV-2 entry to host cells by binding to the ACE2-receptor and spike protein receptor binding domain at IC_50_ values of 59.66 and μg/mL, respectively. Additionally, the results indicated the ability of LF-Zn-NPs to inhibit SARS-CoV-2 replication by interfering with RNA-dependent RNA polymerase “RdRp” activity at IC_50_ of 49.23 μg/mL. In vivo, the LF-Zn-NPs displayed a protective and therapeutic activity against induced pulmonary fibrosis in Bleomycin-treated male albino rats owing to its anti-inflammatory, antioxidant, and significant reduction in CRP, LDH, ferritin, and D-dimer levels. The obtained findings offer a promising route for biosynthesized Zn-NPs and LF-Zn-NPs as promising candidates against COVID-19.

## Introduction

Severe acute respiratory syndrome coronavirus 2 (SARS-CoV-2) is an enveloped RNA virus that causes coronavirus disease 2019 (COVID-19) pandemic with about 7 million deaths to date. Severe infection of respiratory tract cells by SARS-CoV-2 is usually associated with an excessive inflammatory reaction with an uncontrolled surge in the cytokines level (known as cytokine storm) with impaired functions of T and B cells^1^. Consequently, the overexpression of MCP-1, TNF-α, and transforming growth factor (TGF)-β1 and IL-6 contributes to the massive production of reactive oxygen species (ROS) and the development of lung fibrosis^2^. High levels of IL-6 and IL-8 cause intravascular coagulation because of the activation of coagulation and complement cascades. Also, cardiac complications can be caused by direct damage upon virus entry through angiotensin-converting enzyme (ACE) 2 in coronary endothelial cells and cardiomyocytes. The receptor binding domain (RBD) of spike glycoprotein (S)1 is responsible for viral attachment to the host cell surface^3^. Following endocytosis and uncoating, the viral RNA releases into the cytoplasm and translates to generate polyproteins (pp) 1a and 1ab, which are processed, via chymotrypsin-like proteases (3CLpro) and papain-like proteases (PLpro), into 16 non-structural proteins (nsp). Those nsp include nsp4 and nsp6 for double-membrane vesicles formation (as protective microenvironment of RNA replication), RNA dependent RNA polymerase (RdRP; nsp 12) and its cofactors (nsp 7 & nsp8) and helicase (nsp 13) with its cofactor nsp9. And exonuclease (nsp14), methyltransferases (nsp14&nsp16) with its cofactor nsp10 as well as 3CLpro (nsp5) and PLpro (nsp3). RdRP synthesizes complementary negative-strand RNA as a template for the generation of positive-strand RNA, and a set of this sub-genomic mRNA translates into structural proteins (spike, membrane, envelope, and nucleocapsid) forming virions. These nsps, composing the replication-transcription complex, are crucial for viral replication and are therefore promising targets against SARS-CoV2.

Nanotechnology is currently considered as a fundamental component of several technological applications attributed to its exponential development in many areas, including energy, medicine, environment, water, food, and cosmetics sectors, in addition to diverse aspects of daily life applications^4^. Hence, nanoparticles (NPs) are the precursors of the macrostructures of innovative materials with a unique class of materials with several applications^5^. They usually have a larger surface area than their macro-structural analogues, which makes them more chemically reactive^6^. Metallic NPs have attracted the interest of several scientists in the field of nanotechnology owing to their promising outcomes when applied in fields such as electronics, packaging, cosmetics, imaging, infection control, and drug delivery^7^. Among others, zinc oxide nanoparticles (ZnO-NPs) have attracted particular interest because of their distinctive structure, high surface area, and diverse physiochemical properties, making them an appealing option for biological applications^8^. Zinc is an essential trace element that is found in all biological tissues and is an essential part of the majority of enzyme systems. Consequentially, it contributes to the body's metabolism and is absorbed during neuronal cell development, hematopoiesis, and the assimilation of nucleic acids and proteins^9^. ZnO-NPs are more quickly absorbed by biological tissues than zinc metal and are more biocompatible with human cells than other types of nanoparticles.

In addition, a variety of viruses, including SARS-CoV-2 and several respiratory and herpes viruses, are effectively combatted by ZnO-NPs^10^. This is attributed to the potency of zinc to enhance antiviral immunity via the maintenance of cytotoxic T cells, B cells and natural killer cells functions with the suppression of T regulatory cells (Tr) and to inhibit inflammatory mediators (e.g., NF-κB and ROS) generation. Additionally, zinc was reported to strengthens epithelium integrity and decreases thrombosis formations by blocking the aggregation of platelets^1^.

Various natural products and microorganisms have been successfully utilized for green synthesis of Zn-NPs. the biosafety of plant based NPs is well reported, hence Zn-NPs are synthesized by many medicinal plants, such as *Aloe vera*^11^, *Azadirachta indica*^12^, *Passiflora caerulea*^13^ and *Artocarpus heterophyllus*^14^. Accordingly, *Coleus forskohlii Briq extract* was used for green synthesis of Zn-NPs. The *C. forskohlii Briq* plant is widely and mostly distributed in the Mediterranean region^15^. Several studies reported the application of *C. forskohlii* for stomach pains relief, heart diseases treatment, urination and respiratory disorders in addition to hypotension treatment^16^. Additionally several studies reported its wide biological activities including antibacterial, antioxidant, anti-inflammatory, anti-allergic, and anti-cancer properties, that could be attributed to its high contents of polyphenolic compounds (flavonoids and phenolics), terpenoids, tannins, reducing sugars, and alkaloids^17^.

On the other hand, the lactoferrin protein (LF) has antiviral, immunomodulatory, and anti-inflammatory (e.g., lowering TNF-α and IL-6 levels) properties, so it could be a good treatment candidate for severe cases of COVID-19. Both LF and Zn-NPs have a direct effect on nidoviruses replication, to which SARS-CoV2 belongs, by inhibiting RdRP and misfolding replicase polyproteins^18,19^. Both lactoferrin (LF) and Zn-NPs are certified by the Drug Administration as safe components for use by both humans and animals.

Therefore, the current study hypothesized a synergistic antiviral activity for Zn-NPs when coated with LF protein against SARS-CoV-2 infection and induced pulmonary fibrosis. Accordingly, the present study aims to develop a novel nanoformulation of LF-coated Zn-NPs for in vitro evaluation of its inhibitory effect on SARS-CoV2 infection. In addition, evaluating the ability of both biosynthesized Zn-NPs and LF-coated Zn-NPs to alleviate the pulmonary fibrosis in vivo using Bleomycin-induced lung fibrosis in adult males’ albino rats.

## Results and discussions

### Lactoferrin purification

Since whey proteins are well known as promising antiviral candidates, it is crucial to protect them from the body's barriers, improve their stability and increase their bioactive functions through nanofabrications using NPs like nanometals^20,21^. LF is a glycoprotein with multi-biological properties such as antibacterial, antioxidant, antiviral activities^22–24^. The generation of new NPs-protein complexes as a result of protein interaction or adsorption on the surface of NPs is thought to be the source of NPs' bio-reactivity. The newly created complexes have the ability to alter the overall bioactivity of the newly produced NPs by altering the structural conformation of the proteins adsorbed on their surface. Additionally, the surface of NPs can alter and modify the structure of the adsorbed protein, changing the protein's primary function^25^. Here, bovine LF was eluted from a Mono S column using NaCl gradient after applying skim milk to equilibrated column by Tris HCl buffer, pH 8.0. All fractions containing LF were pooled separately, concentrated and fractionated by Sephacryl S100 column. LF has a potential activity against a wide range of viral infections via postponing the viral entry into the host cells either by blocking the cell receptor or quenching the virus particles^26,27^. This approach has been identified for several coronaviruses by blocking the HSPGs surface protein in vitro, which is a critical co-receptor for COVID-19 entry^28^. LF also exhibited significant immunomodulatory and anti-inflammatory activities^29^, which may help to explain why it is included in some COVID-19 treatment regimens. This anti-inflammatory mechanism of LF based on its ability to regulate the expression of pro-inflammatory genes within the nucleus of the host cells^30^.

### Green synthesis of Zn-NPs through *Coleus forskohlii* Briq extract

A combination of biomolecules, including enzymes, proteins, and peptides, as well as phytochemicals derived from plants, is frequently used for the biosynthesis of metallic NPs with various morphologies and sizes^31,32^. This biogenic approach has recently shown to be effective for producing nanomaterials with a variety of morphologies. Our research focuses on improving the biosynthesis of Zn-NPs using through *C. forskohlii* Briq. Biogenic reduction of zinc salt to Zn-NPs via exposure to ethanolic extract of the plant leaf was processed by a color change to be a greenish color after freeze-drying process (Fig. S1). Green synthesis approach depend on a spontaneous reduction of zinc ions (Zn^2+^) in the presence of ammonium hydroxide (NH_4_OH) forming zinc hydroxide (Zn(OH)_2_), then followed by synthesis of Zn-NPs upon heating. The yield of the obtained Zn-NPs was 7.2 g, which indicates about 72% of the precursor utilized was converted into pure Zn-NPs.

### Functionalization of Zn-NPs with purified LF (LF-Zn-NPs)

The green synthesized Zn-NPs were functionalized with the purified LF at different concentration with the biosynthesized Zn-NPs. The results indicated a reduction in the Zn-NPs binding at higher LF concentrations, whereas the binding percentage reduced to 89.57%, 83.09%, 69.46%, 56.21%, and 52.34% with LF at different concentrations of 1.2, 1.4, 1.6, 1.8 and 2.0 mg/mL, respectively. In addition, the results showed that 1 mg of LF was bound to about 97.34% of Zn-NPs (0.5 mg/mL). In order to determine the maximum (saturated) concentration of Zn-NPs on LF protein, the ideal dose (1.0 mg/mL) of LF was incubated with successive doses of Zn-NPs, with the unbound Zn-NPs being separated by the centricon. It was shown that 100% of the NPs were chelated by LF at 0.4 mg/mL of Zn-NPs.

### Characterization of the prepared Zn and LF-Zn-NPs

**Figure 1:**
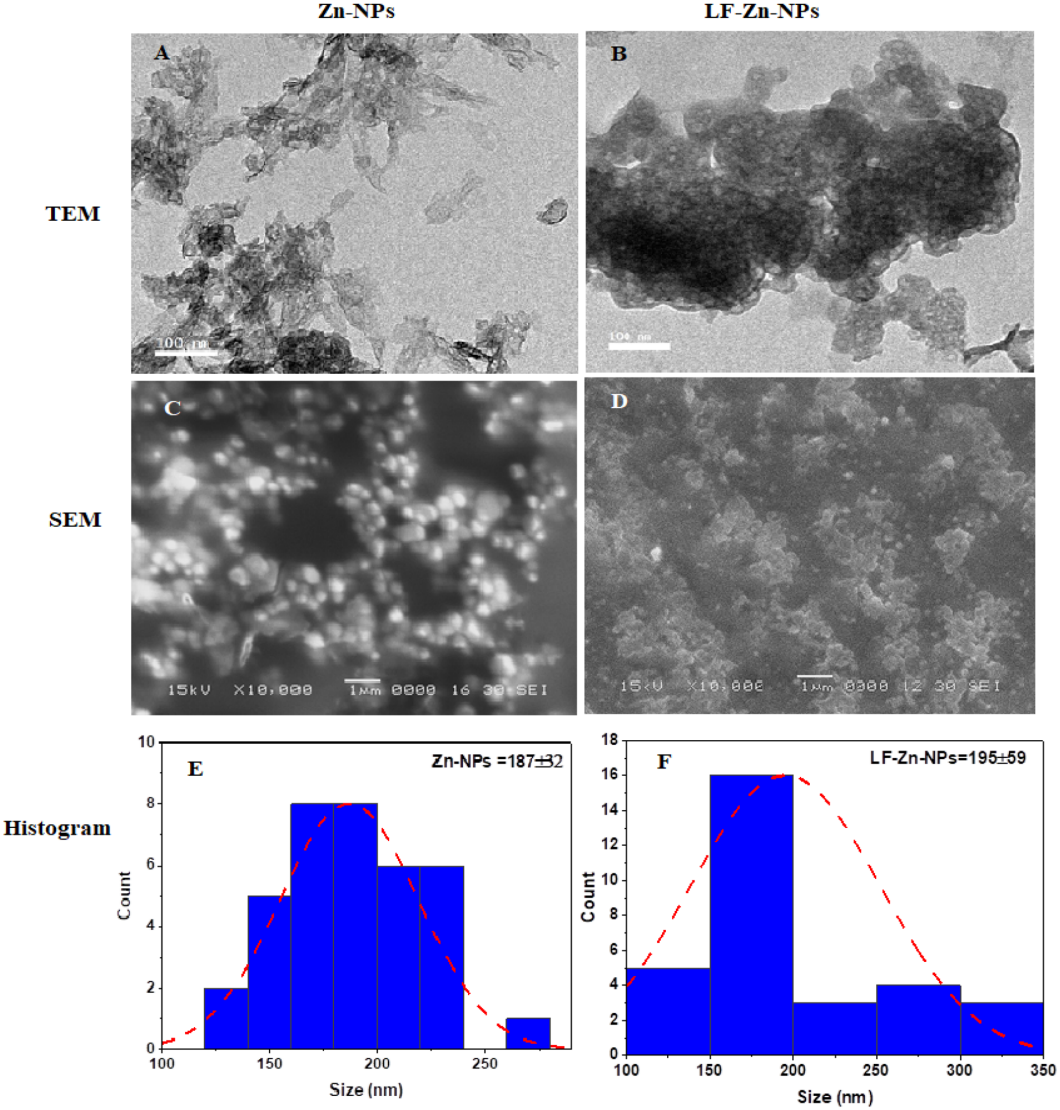
The transmission electron micrographs (TEM) of the prepared NPs were included for Zn-NPs (**A**) and LF-Zn-NPs (**B**). The scanning electron micrographs (SEM) of the Zn-NPs (**C**) and LF-Zn-NPs (**D**) at ×10000. Histogram analysis of.

Different instrumental analysis was applied for characterizing the prepared Zn-NPs and confirming the functionalization of the NPs surface with purified LF protein. TEM analysis (Fig. 1A,B) revealed condense and compacted spherical shaped structure of Zn-NPs with organic layer surrounding the Zn-NPs in the case of LF-Zn-NPs, which could be attributed to capping protein^33^. SEM results confirmed the TEM results, whereas the Zn-NPs indicated condensed oval to spherical shaped in multi-growth phases and sizes (Fig. 1C). Some particles upper unregular and compacted, which could be attributed to the heavily prepared specimens^34^. The prepared LF-Zn-NPs revealed the same spherical to oval shaped structure with slightly larger particles (Fig. 1D). The average size of the prepared NPs was obtained by histogram analysis for the SEM image taken by ImageJ software. The average size of Zn-NPs and LF-Zn-NPs were 187 nm and 195 nm, respectively (Fig. 1E and 1F). On the same regard, the EDX spectrum (Fig. S2A) of Zn-NPs confirmed the presence of intense signal of Zn (81.93%) with low signal for oxygen (18.07%) at their corresponding surface energies 8.630 and 0.525 keV, respectively. However, the EDX spectrum of LF-Zn-NPs (Fig. S2B) confirmed the elemental distribution of Zn more than O_2_ with composition of 69.59% and 30.41%, respectively.

**Figure 2:**
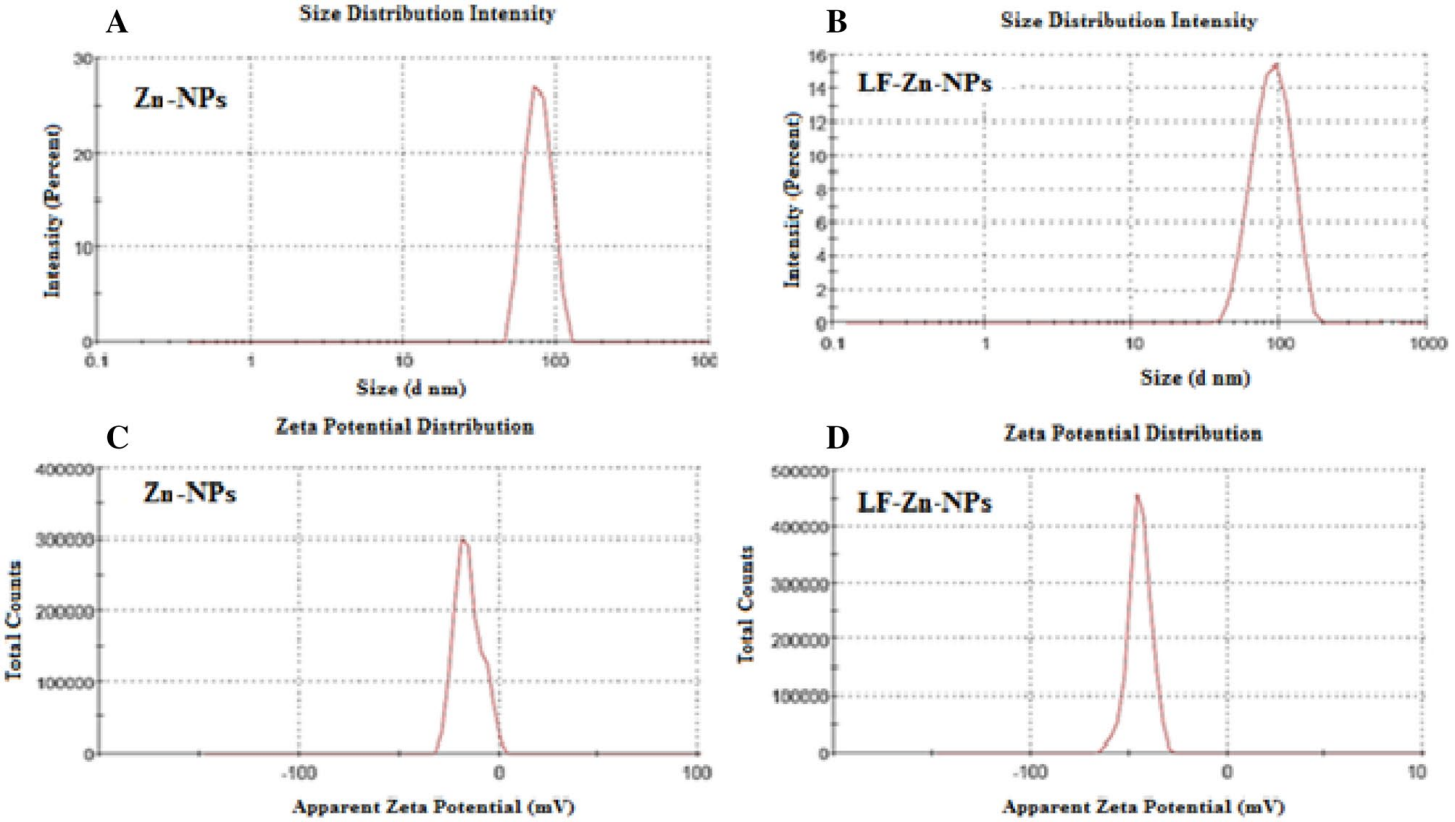
The hydrodynamic sizes of the prepared Zn-NPs and LF-Zn-NPs (**A**,**B**, respectively) with corresponding zeta-potentials (**C**,**D**, respectively).

Furthermore, the hydrodynamic size and zeta-potentials for the green synthesized Zn-NPs were 77 ± 5.50 nm and − 20.25 ± 0.35 mV, respectively (Fig. 2A,C). The results indicated the ability of *Coleus forskohlii Briq* extract to reduce zinc nitrate salt to negatively charged particles in the nanoscale. On the other hand, the prepared LF-Zn-NPs revealed increased size of about 98 ± 6.40 nm with significant reduction of surface charge to about − 44.3 ± 3.25 mV (Fig. 2B,D). The difference in the average size of Zn-NPs and LF-Zn-NPs obtained by zeta sizer analysis (77 nm and 98 nm) and SEM result (187 nm and 195 nm) might because SEM images were captured for very small portion of the prepared samples. The negative charge of the biosynthesized Zn-NPs NPS could be attributed to different functional groups loaded in the NPs surfaces from the applied plant extract^35^. The size increase and zeta-potential reduction are strong evidences for functionalization of LF protein on the Zn-NPs surfaces and in accordance with other studies^36,37^. Additionally, several studies reported the importance of such protein layer in increasing the NPs stability and biocompatibility in the biological fluids^20^.

**Figure 3:**
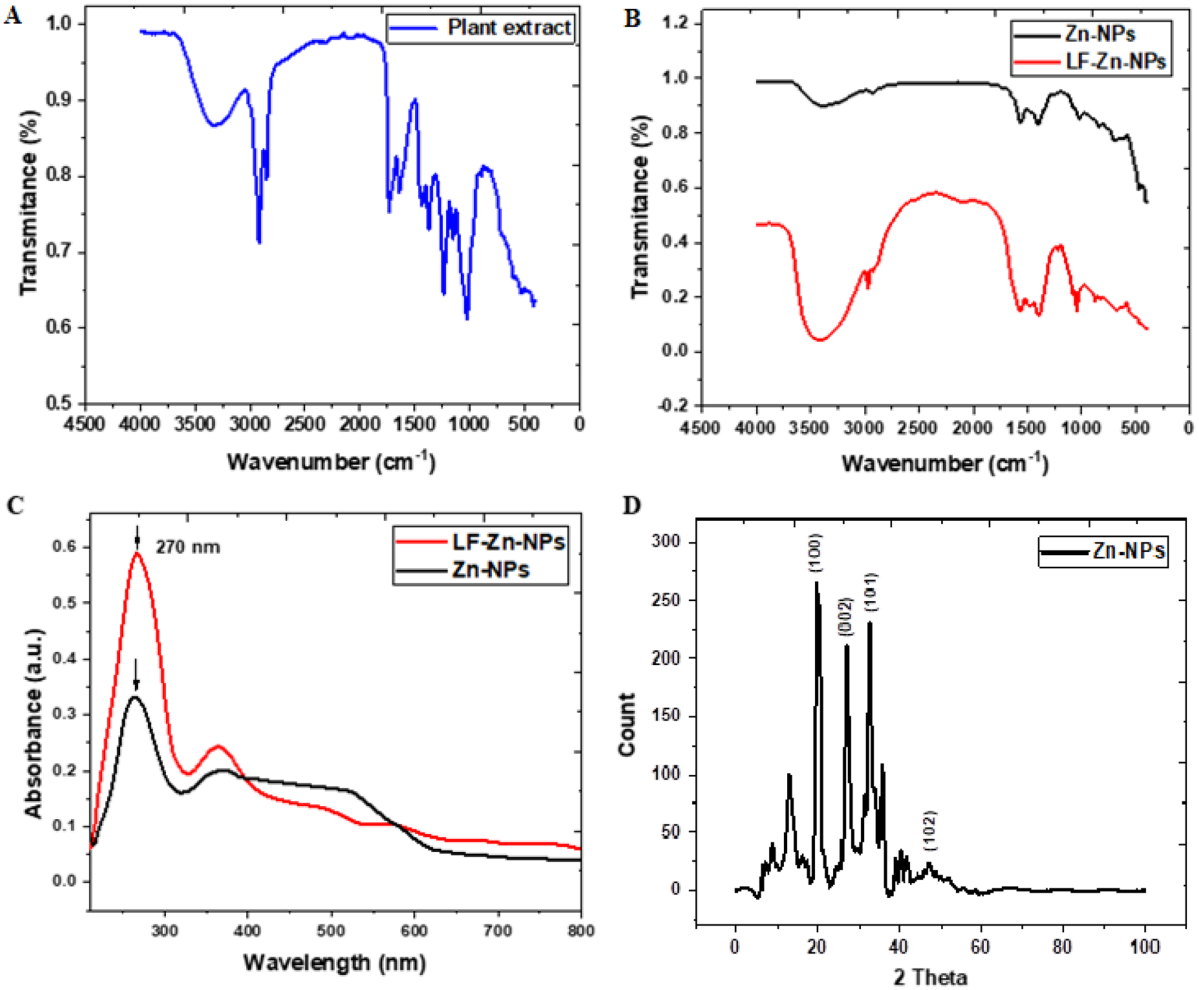
(**A**) FT-IR spectra of the *Coleus forskohlii* Briq plant extract revealing its functional groups. (**B**) FT-IR spectra of the prepared Zn-NPs (Blue curve) and LF-Zn-NPs (Red curve). (**C**) UV spectra of the prepared Zn-NPs and LF-Zn-NPs, where (**D**) is the X-ray diffraction charts (XRD) of the prepared Zn-NPs.

The functional groups in the plant extract and the prepared NPs were elucidated through FT-IR analysis. The results (Fig. 3A) revealed diverse number of functional groups in the plant extract including O–H groups of alcohols and polyphenolic compounds detected at range 3320–3000 cm-^1^^38^. The stretching vibration of alkane (C–H), carbonyl groups (C=O) were also indicated at 2917 and 1639 cm^−1^, in addition to N–H bending of amine and amid groups at 1433 cm^−1^^39,40^. Other functional groups were also elucidated at 1023 cm^−1^ (indicated C-O vibration, N–H, and C–H) and 855 cm^−1^ (assigned to C=CH_2_ vibration). Similarly, the green synthesized Zn-NPs also revealed several functional groups, as indicate in Table S1 (Supplementary material), which is most likely to be related to the plant extract functional groups. Several studies reported the adsorption of diverse functional groups on the plant mediated green NPs^13,41^. And indicated their significant roles in enhancing the NPs stability^40,42^. Interestingly, the prepared LF-Zn-NPs revealed larger numbers of functional groups on the NPs surface with a slight shifting for all groups to higher wavenumbers (Fig. 3B), which confirms protein loading in the Zn-NPs surfaces. Figure 3C shows the UV–VIS spectral analysis of the prepared Zn-NPs revealed a distinct band at 270 nm, which confirmed the ability of *C. forskohlii* leaves extract to reduce Zn ions to Zn-NPs and in accordance with the ZnO-NPs prepared by *Punica granatum L* extract^43^. Contrary, other studies reported plant-mediated Zn-NPs in different UV–Vis ranges (between 250 and 370 nm), which are largely related to NPs sizes^35,44^. The LF-coated Zn-NPs revealed a higher intensity peak at the same wavelength (270 nm), which could be attributed to the detection wavelength of LF protein, as similarly reported in the interaction of ZnO-NPs and bovine serum albumin^45^. The phase identity, crystalline nature, and purity of the biosynthesized zinc NPs were analyzed by XRD (Fig. 3D). The obtained results showed four distinct intense peaks belong to Bragg’s reflection 101, 002, 101 and 103 have appeared, which correspond to zero-valent Zn (JCPDS PDF #00-004-0831) in the biosynthesized Zn-NPs.

### Internalization of free LF in comparison to Zn-NPs and LF-Zn-NPs

**Figure 4:**
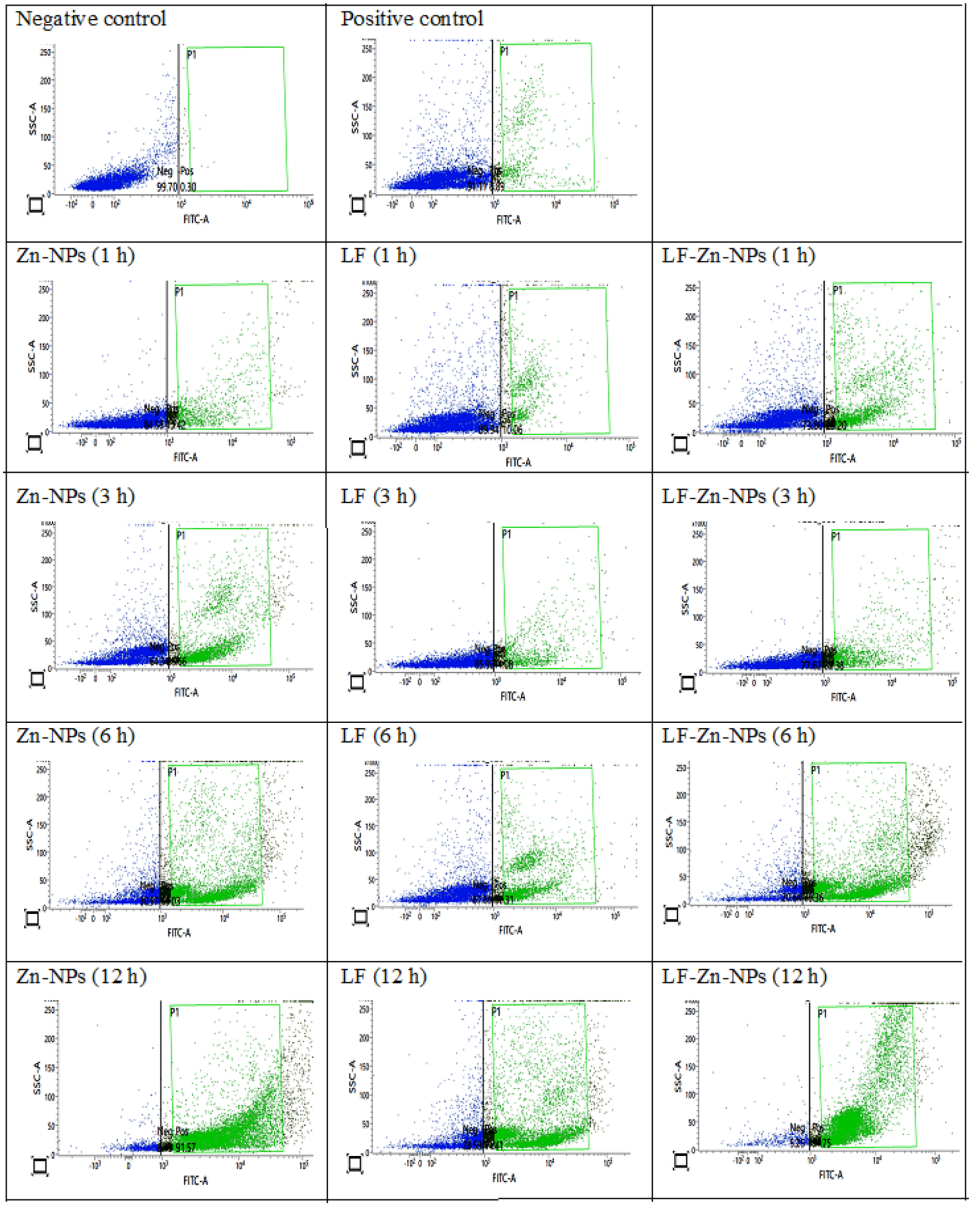
Flow cytometry dot charts of the cell internalization of 100 mg/mL of Zn-NPs, LF-Zn-NPs, and free LF tagged with FITC, at different time intervals (1, 3, 6, and 12) using PBMCs.

The internalization characteristics of Zn-NPs, LF, and LF-Zn-NPs indicate their bio-distribution and transport effectiveness. In this investigation, flow cytometry was used to monitor the cellular uptake of free and NPs compounds in PBMCs at various time intervals (Fig. 4). The results of this research demonstrated that the amount of time that complexes are incubated with cells has a significant impact on their ability to Zn-NPs uptake. Compared to LF alone, the prepared LF-Zn-NPs demonstrated superior cellular absorption. These results showed that Zn-NPs promote and enhance LF internalization and cell uptake over time.

### Assaying of the cytotoxicity of LF-Zn-NPs on normal cells

**Figure 5:**
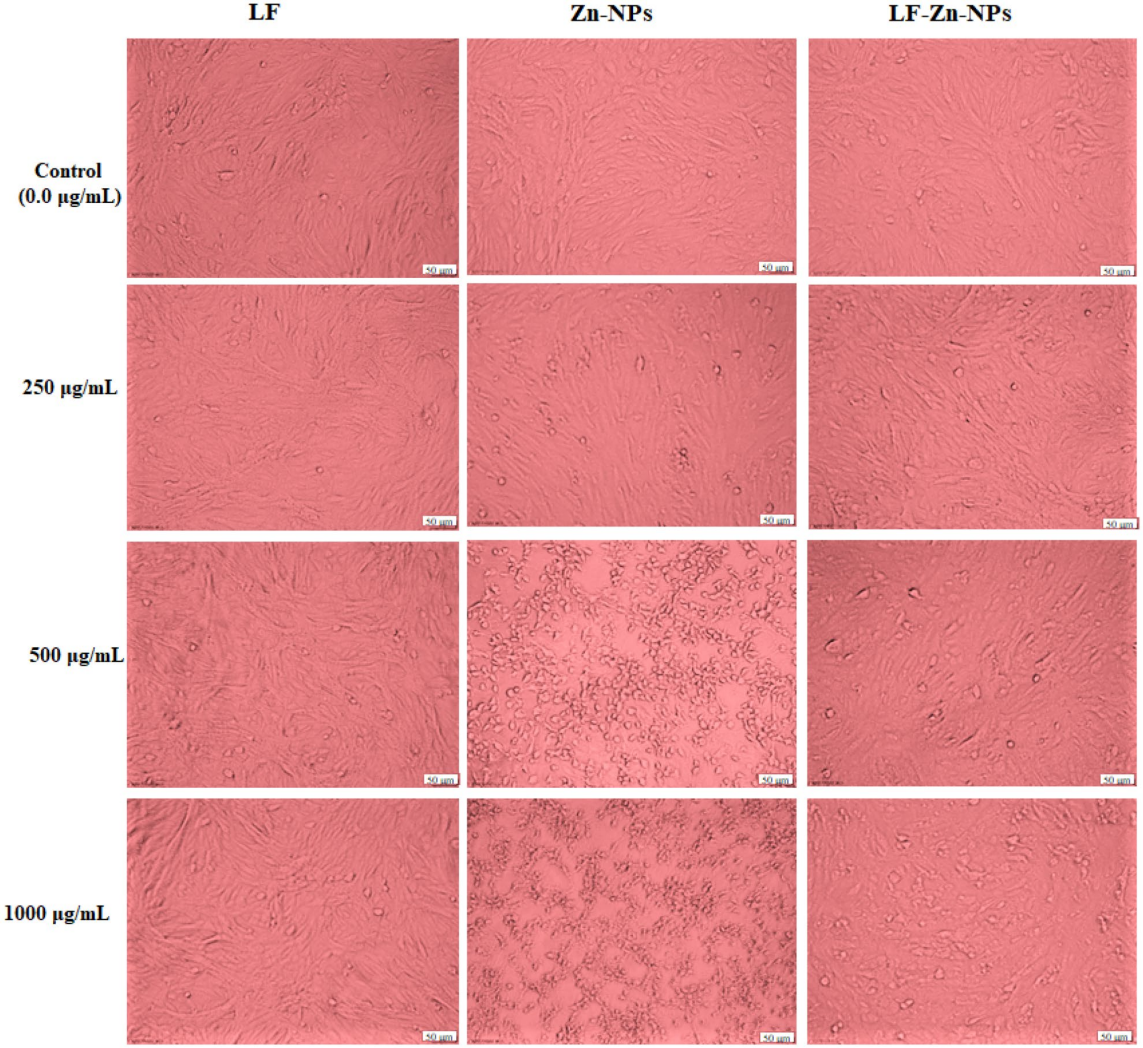
Effects of the biosynthesized NPs on morphological modifications of normal Vero cells as observed under phase contrast microscope. Vero cells were exposed to the prepared Zn-NPs and LF-Zn-NPs at different concentrations of 250 μg/mL, 500 μg/mL, and 1000 μg/mL. The scale bar in all images is 50 µm and magnification 10X.

The cytotoxicity of the biosynthesized Zn-NPs and the prepared LF-Zn-NPs was determined in vitro against both Vero cells and PBMCs cells as compared to free LF. The results revealed that coating of the biosynthesized Zn-NPs with LF was significantly increase their safety on the treated Vero cells, compared to Zn-NPs (Fig. 5). Table 1 shows that the highest EC_100_ values were recorded for LF followed by the prepared LF-Zn-NPs, whereas free Zn-NPs showed the lowest EC_100_ and IC_50_ values against both treated cells. The IC_50_ values on Vero cells were determined to be 1352, 226.71 and 985 μg/mL for LF, Zn-NPs and LF-Zn-NPs, respectively. However, IC_50_ values on PBMCs were determined to be 1138, 165.17 and 916.29 μg/mL, respectively.

**Table 1:** EC_100_ and IC_50_ (μg/mL) and SI values of the biosynthesized Zn-NPs and LF-Zn-NPs samples against Vero and human normal (PBMCs) cell lines.

Cell lines	value	LF	Zn-NPs	LF-Zn-NPs
Vero	EC_100_	67.57 ± 3.74	7.53 ± 0.57	47.46 ± 4.58
	IC_50_	1352 ± 97.43	226.71 ± 18.61	985 ± 57.36
PBMCs	EC_100_	87.59 ± 7.53	11.36 ± 0.85	53.35 ± 3.66
	IC_50_	1138 ± 76.29	165.17 ± 12.93	916.29 ± 81.38

All values were expressed as mean ± SEM.

### In vitro anti-SARS-CoV-2 application of the prepared Zn-NPs and LF-Zn-NPs

**Figure 6:**
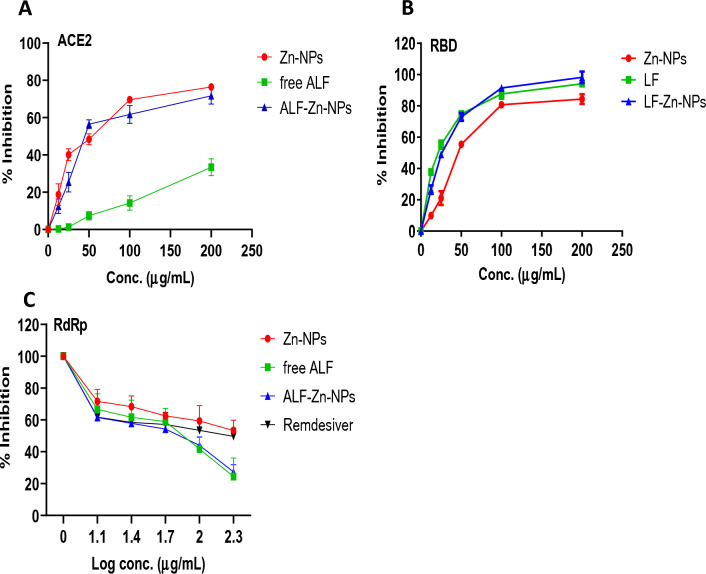
Evaluation of the ACE2-SARS-CoV-2 spike inhibition (**A**), the RBD-SARS-CoV-2 neutralization effect (**B**), and the inhibitory effect on RdRp-SARS-CoV-2 (**C**) for Zn-NPs, LF and LF-Zn-NPs at different concentrations (25–200 µg/mL). Results are expressed as mean ± standard error. p < 0.05. Data are the mean ± SE, n = 3.

The inhibition of the SARS-CoV-2 was evaluated in vitro using the ACE2/spike inhibitor screening kit through inhibition of the ACE2/spike interaction. The results (Fig. 6A) indicated a dose-dependent inhibition of ACE2/spike with increasing the NPs concentrations from 12.5 to 200 mg, whereas the maximum inhibition was about 33, 76.5, and 71.7% from LF, Zn-NPs, and LF-Zn-NPs, respectively at 200 mg concentration. The IC_50_ values were determined to be 318.40 ± 2.13, 47.72 ± 2.53, and 59.66 ± 3.41 µg/mL for LF, Zn-NPs and LF-Zn-NPS, respectively. Furthermore, the ability of the prepared Zn-NPs and LF coated Zn-NPs to neutralize the RBD domain was evaluated in vitro as compared to free LF. The results revealed significant inhibition ability of the prepared NPs to RBD domain that slightly enhanced with LF coating (Fig. 6B). The results indicated dose-dependent inhibition with maximum activity of about 94, 84, and 98% inhibition through LF, Zn-NPs, and LF-Zn-NPs, respectively. The RBD of the SARS-CoV-2 viral spike glycoprotein interacts with human ACE2 with great affinity^46^. Spikes are the main virulent factor of SARS-CoV-2, and all research efforts have been directed at identifying compounds that can interact with Spike and therefore prevent SARS-CoV-2 infection^47^. The spike protein is a 1273-amino acid single-pass transmembrane protein with a transmembrane helix, a long N-terminal ectodomain exposed on the surface of the virus, and a short C-terminal tail on the inside of the virus. Piacentini et al., found that in concentrations that are comparable to the physiological ones found in human milk (1–10 M), human LF (hLF) is effective at inhibiting the RBD–ACE2 interaction in solution^46^. They also demonstrated that hLF may bind the ACE2 receptor ectodomain with a high affinity without binding to the RBD^46^. In agreement with our findings, in-silico^48^ and experimental studies^47^ discovered that LF is a promising candidate for preventing SARS-CoV-2 infection due to its ability to bind to the RBD C-terminal domain. The outcomes of molecular docking between LF and various Spike variants clearly imply that single-point mutations in different SARS-CoV-2 variants do not affect LF’s capacity to interact with Spike. This finding lends support to the use of LF in the early stages of SARS-CoV-2 infection^47^. The RdRp is essential enzyme that directly involved in RNA synthesis for SARS-CoV-2 virus. Hence, the effect of the prepared NPs on SARS-CoV-2 replication was evaluated through RNA-dependent RNA polymerase commercialized fluorescence kit as compared to Remdesivir as standard RdRp inhibitor. As shown in the results (Fig. 6C), the free LF and LF-Zn-NPs were superior in targeting and inhibiting the RdRp activity with 76- and 73% inhibition at concentration 200 µg/mL compared to Remdesivir (about 50% inhibition at the same conc.). On the other hand, the maximum RdRp inhibition by Zn-NPs was about 47% (200 µg/mL), which indicated the essential role of LF in targeting RdRp activity, which is in line with other studies^49,50^. The significant ability of LF-Zn-NPs to inhibit RdRp even more than the standard RdRp drug (Remdesivir) represents a promising and safer alternative for post-infection treatment not only for SARS-CoV-2 but for many RNA viral infections^49,51^.

### The predicted inhibitory mechanism of lactofferin on the entry and replication of SARS-CoV-2

**Figure 7:**
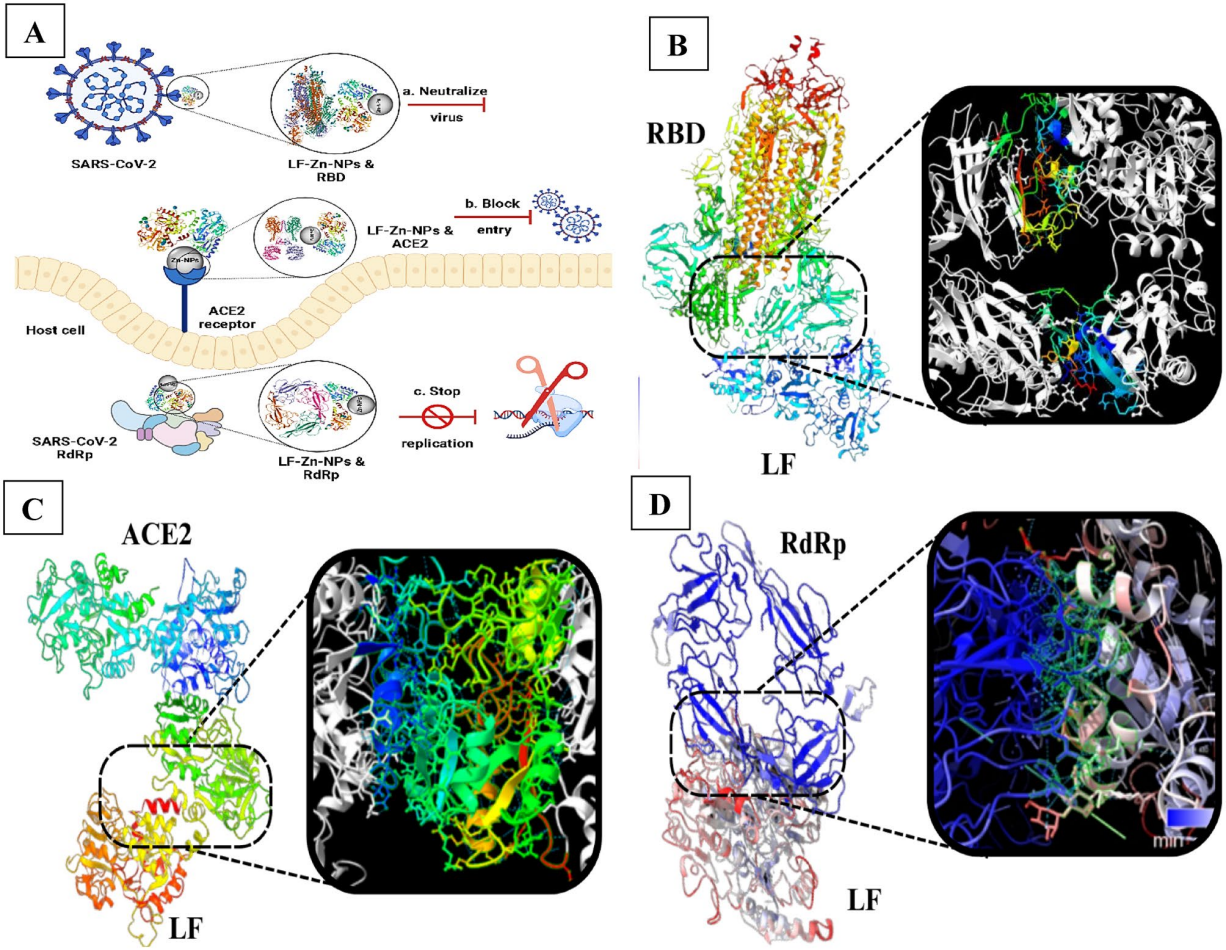
(**A**) Schematic illustration of the antiviral mechanism of action of LF-Zn-NPs against SARS-CoV-2 by binding of LF to viral spike and neutralize virus effect, binding of Zn-NPs to ACE2 receptors and block viral entry, and binding to RdRp and stop viral replication. (**B**) Protein docking results of bovine lactoferrin (1BLF) and SARS-CoV-2 RdRp (6VYO), score 18928, 309 H-bond, and lowest energy − 1338.3; (**C**) bovine lactoferrin (1BLF) and human ACE2 (6M1D), score 21438, 278 H-bond, and lowest energy − 839.5; and (**D**) bovine lactoferrin (1BLF) and SARS-CoV-2 RBD (7KJ5), score 24510, 200 H-bond, and lowest energy − 979.3.

In general, LF possesses potent antiviral efficacy against both DNA and RNA viruses that usually exploit common cellular receptors to invade the host cells. These receptors, including ACE2, provide anchoring sit on the host cell surface and recognized by SARSCoV-2 to invade the cells. Zhou et al. (2020) demonstrated that the entry of SARS-CoV-2 to host cells is mediated by ACE2 via fusion between host cellular membranes and viral particles. Also, Piacentini et al.^46^ demonstrated LF has high affinity to interact with ACE2 receptor and appears to directly interfere with RBD–ACE2 binding, which competes with virus for receptor occupancy, consequently interfere with virus cell entry. Moreover, LF strongly inhibits the activity of SARS-CoV-2 RdRp whose inhibition arrest viral replication^18^. Molecular docking plays a pivotal role within the drug development continuum. In this context, its application was undertaken to evaluate the hypothesized binding affinities of LF with the SARS-CoV-2 RdRp, RBD, and human ACE2. The objective was to identify the potential protein candidate exhibiting optimal efficacy. Notably, the interaction between LF and the viral RdRp exhibited the most pronounced affinity, followed by RBD and ACE2 (Fig. 7A–D). This investigation sheds light on potential avenues for enhancing therapeutic interventions against the virus.

### In vivo anti-fibrotic activity on IPF-modeled albino rats

The IPF is a chronic, progressive, and irreversible lethal lung disease, and the annual incidence of IPF is rising, whereas the medical filed still lacks safe and effective therapeutic regimens in the clinic^52^. The Bleomycin-induced animal model of PF is widely used to investigate new anti-fibrotic compounds^53,54^. In the current study, a significant drop was noticed in all hematological measurements including Hb, HCT levels; RBC, with significant increase in WBCs, and platelets count; as a consequence, to BLM intoxication in compare to the healthy group, which are in accordance with^55,56^. BLM is known to binds with Fe^2+^, thereby causing iron deficiency and anemia, which directly and/or indirectly induces the ROS oxidative stress in red blood corpuscles^57,58^. Favorably, administration of IPF rats with Zn-NPs and LF-Zn-NPs resulted in a marked improvement in the mentioned hematological parameters close to the healthy values as indicated in Table 2.

**Table 2:** Effect of Zn-NPs, and LF-Zn-NPs treatments on the levels of whole blood biomarkers as compared to non-treated Bleomycin induced PF (positive control) and non-treated healthy rats (as negative control).

Hematological parameters	Control	Non-treated	BLM-treated	
		BLM	Zn-NPs	LF-Zn-NPs
Hb (g/dL)	13.1 ± 0.53	10.82 ± 0.18*	13.6 ± 0.56^#^	14.8 ± 0.19^#^
HCT (%)	39.6 ± 1.7	31.5 ± 0.55*	49.24 ± 1.8^#^	42.44 ± 0.60^#^
RBC (10^6^/uL)	6.4 ± 0.2	4.7 ± 0.32*	7.56 ± 0.24^#^	8.56 ± 1.21^#^
WBC (10^3^/ uL)	6.88 ± 0.88	14.33 ± 2.7*	8.6 ± 0.78^#^	8.1 ± 0.78^#^
PLT (10^3^/uL)	914 ± 52	1020 ± 79*	902 ± 42.6^#^	896 ± 35.36^#^

Data are presented as mean ± standard error; data were subjected to one-way ANOVA followed by post hoc (Tukey) test at p ≤ 0.05. (*) is significantly different from control group; (#**)** is significantly different from Bleomycin group; Zn-NPs: Zinc nanoparticles; LF-Zn-NPs: Lactoferrin coated Zinc nanoparticles; BLM: Bleomycin.

**Figure 8:**
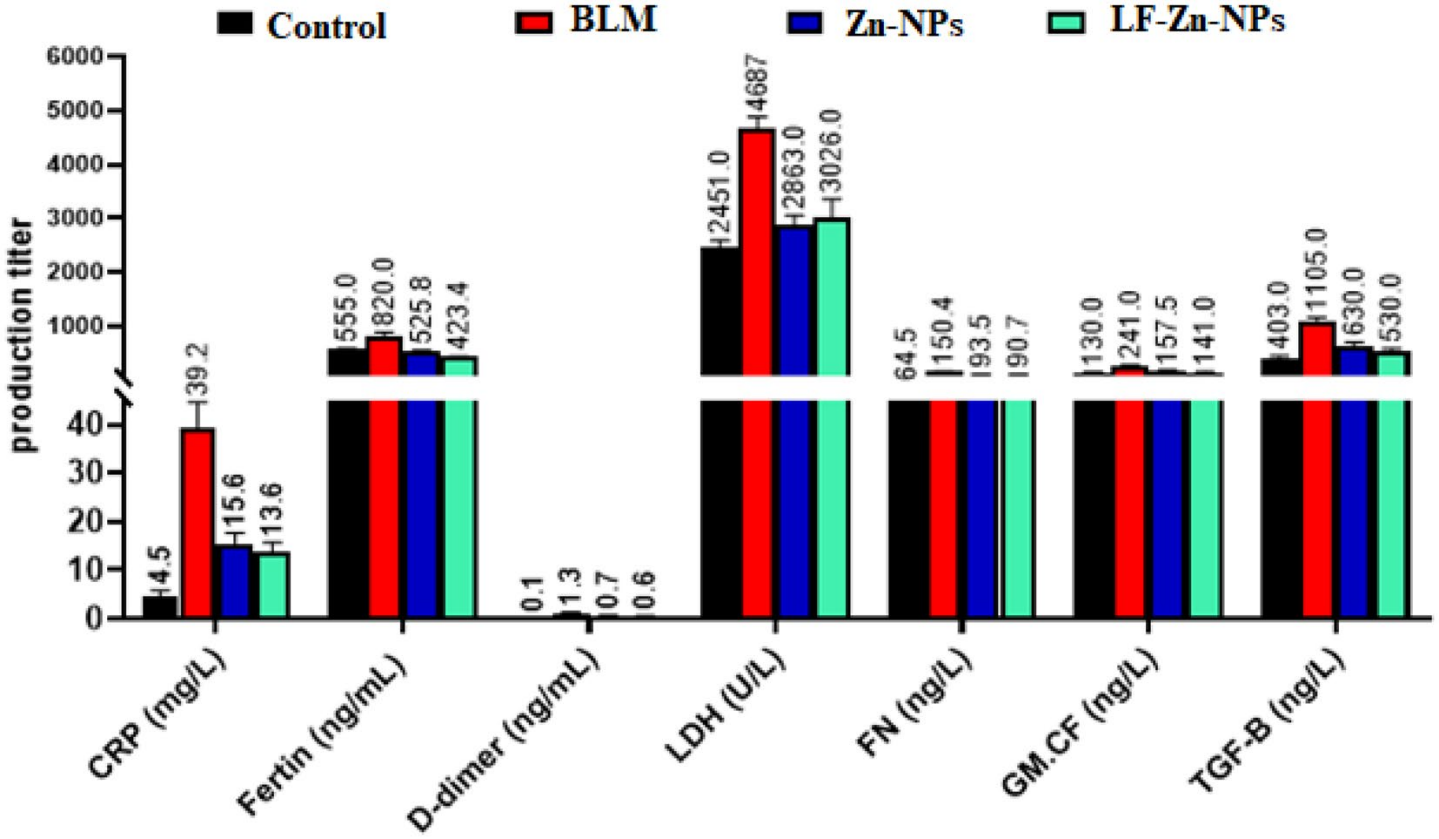
Serum CRP, ferritin, D-Dimer, LDH, FN, GM-CSF, and TGF.β of control, non-treated pulmonary fibrosis (BLM), and pulmonary fibrosis-treated rats with Zn-NPs (Zn-NPs) and Lactoferrin-coated Zinc nanoparticles (LF-Zn-NPs).

Furthermore, the IPF-modeled animals revealed a significant increase in levels of ferritin, CRP, D-Dimer, GM-CSF, FN, LDH, and TGF-β compared with the control group, which is in accordance with the studies of Mehdizadeh et al.^59^ and Ju et al.^60^. Interestingly, treatment of IPF-modeled rats with Zn-NPs and LF-Zn-NPs significantly improved the levels of the mentioned lung biomarkers close to that of control levels (Fig. 8). Furthermore, when compared to the control group, rats with induced IPF showed severe abnormalities in the oxidative status of the lung tissues, as shown by the marked decrease in antioxidant enzyme values (GSH, SOD, CAT, and GPx) along with an increase in lipid peroxidation (MDA) and NO levels. Uncontrolled oxidative stress is among the mechanisms by which BLM cytotoxicity causes lung tissue harm^61^. The BLM compound has the ability to bind Fe (II) ions, creating a complex that is then oxidized to Fe (III) in the presence of O_2_, which reducing oxygen to free radicals and producing reactive oxygen species (ROS), including superoxide, hydroxyl radicals, and Fe (III). Then, this Bleomycin complex forms a nucleophilic interaction with the DNA helix, causing DNA strand breaks and/or membrane lipid peroxidation and subsequent damage to the membrane take place. Noteworthy, Zn-NPs and LF-Zn-NP application for treatment of IPF-modeled rats led to a considerable normalization of the lung GSH content with augmentation of the antioxidant enzymes activity in terms of CAT, GPx, and SOD. Additionally, the ability of Zn-NPs and LF-Zn-NPs to reduce lung MDA and NO levels in comparison to the equivalent values of the group of animals with IPF modelling revealed significant results (Table 3). In this case, we propose that Bleomycin induce the intracellular formation of ROS that negatively affected the cell membrane and organelles, which causes an increase in the MDA level. Hence, the lactoferrin-coated Zn-NPs inhibit this excessive level of ROS which decreases the rate of MDA detected^62^. The antioxidant and anti-inflammatory effects of lactoferrin-coated Zn-NPs was confirmed by a decreased the MDA level, NF-κB, and proinflammatory cytokines, in addition to increasing the reduced GSH level, and attenuated lung injury by histopathological analysis^63^.

**Table 3:** Lung oxidant-antioxidant markers of control, pulmonary fibrosis-modeled and pulmonary fibrosis-treated rats.

Oxidative stress markers and enzymes	Control	Non-treated	BLM-treated	
		BLM	Zn-NPs	LF-Zn-NPs
NO (μmol/g)	10.1 ± 0.47	25.2 ± 2.1*	15.6 ± 4.5^#^	11.6 ± 5.6^#^
MDA (μmol/g)	427 ± 28	887 ± 43*	604.3 ± 36.2^#^	515.5 ± 16.5^#^
GSH (nmol/g)	33.2 ± 2.7	12.11 ± 1.2*	26.5 ± 4.9^#^	29.32 ± 7.2^#^
SOD (U/g)	3.2 ± 0.06	1.4 ± 0.11*	2.6 ± 0.12^#^	2.95 ± 0.14^#^
GPx (U/g)	1264 ± 71	469 ± 28*	925.3 ± 31.5^#^	1102 ± 84.2^#^
CAT (U/g)	25 ± 1.8	10.4 ± 1.2*	18.6 ± 2.4^#^	23.2 ± 5.1^#^

Data are presented as mean ± standard error; data were subjected to one-way ANOVA followed by post hoc (Tukey) test at p ≤ 0.05. (*) is significantly different from control group; (#**)** is significantly different from Bleomycin group; Zn-NPs: Zinc nanoparticles; LF-Zn-NPs: Lactoferrin coated Zinc nanoparticles; BLM: Bleomycin.

**Figure 9:**
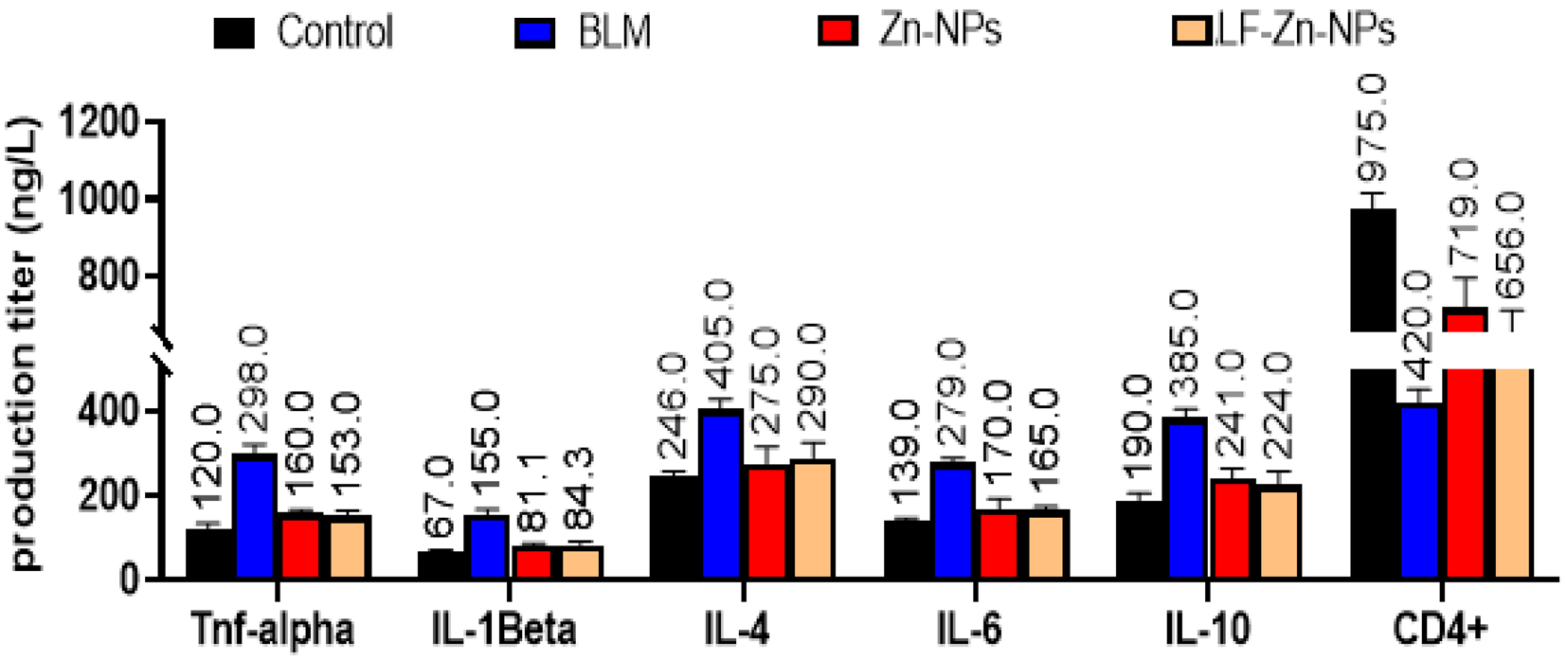
Serum TNF-α, IL-1β, IL-4, IL-6, IL-10, and CD4+ of the control group, non-treated pulmonary fibrosis-induced group (BLM), and pulmonary fibrosis-treated rats with Zn-NPs (Zn-NPs) and Lactoferrin-coated zinc-nanoparticles (LF-Zn-NPs).

In the current study, the IPF-modeled rats had significantly higher blood levels of the pro-inflammatory cytokines TNF-, IL-1, IL-4, IL-6, and IL-10 and significantly lower CD4+ levels than the normal rats' group, which are in line with the results reported by Gul et al.^64^ and Mehdizadeh et al.^59^. Fortunately, injection of Zn-NPs and LF-Zn-NPs effectively reversed these lung fibrosis deteriorations in the group of IPF-modeled rats (Fig. 9). BLM-induced toxicity and ROS accumulation are associated with the strong inflammatory consequences. This inflammation leads to influx of neutrophils (1^st^ line of defense) into the alveoli (pneumonitis) within 3–7 days and chemotaxis of macrophages and lymphocytes in to the alveoli within 6–30 days, which finally resulted in TLC increasing in the BLM group. Lactoferrin-coated Zn-NPs can be absorbed through the digestive tract into the lungs, protecting the lungs against accumulative oxidative stress. Furthermore, studies in humans and animals have proved that hyperoxia negatively alters the innate immunity and amplified the risk of pneumonia^65,66^. Furthermore, LF can be used as a natural agent for the selective decontamination of the digestive tract (SDD) in order to prevent infection in the blood and even in the lungs^67^. All these evidence implies that LF-Zn-NPs is a novel agent for alleviating of systemic oxidative stress, VILI, and even ventilator-associated pneumonia in the ICU. The anti-inflammatory effect of LF-Zn-NPs is mainly attributed to its noteworthy role in the induction of anti-inflammatory cytokines interleukin (IL)-4, (IL)-6 and IL-10, and reductions in the pro-inflammatory cytokines tumor’s necrosis factor-α and IL-1β by suppressing NF-κB signaling pathway^68^. Additionally, it has been noted previously that LF-coated Zn-NPs encourage the macrophage transition from an inflammatory to a tolerogenic phenotype, which is essential for tissue homeostasis^69^. Apoptosis induction by uncontrolled oxidative stress can also be lessened by LF-coated Zn-NPs by lowering intracellular levels of ROS^70^. Moreover, the antioxidant activity of LF-coated Zn-NPs is probably related to their capacity to iron scavenging and inhibition of iron-catalyzed formation of ROS^71,72^. Furthermore, LF-coated Zn-NPs augments the proliferation, differentiation, maturation, migration, and function of immune cells^29^.

### Histopathological study

**Figure 10:**
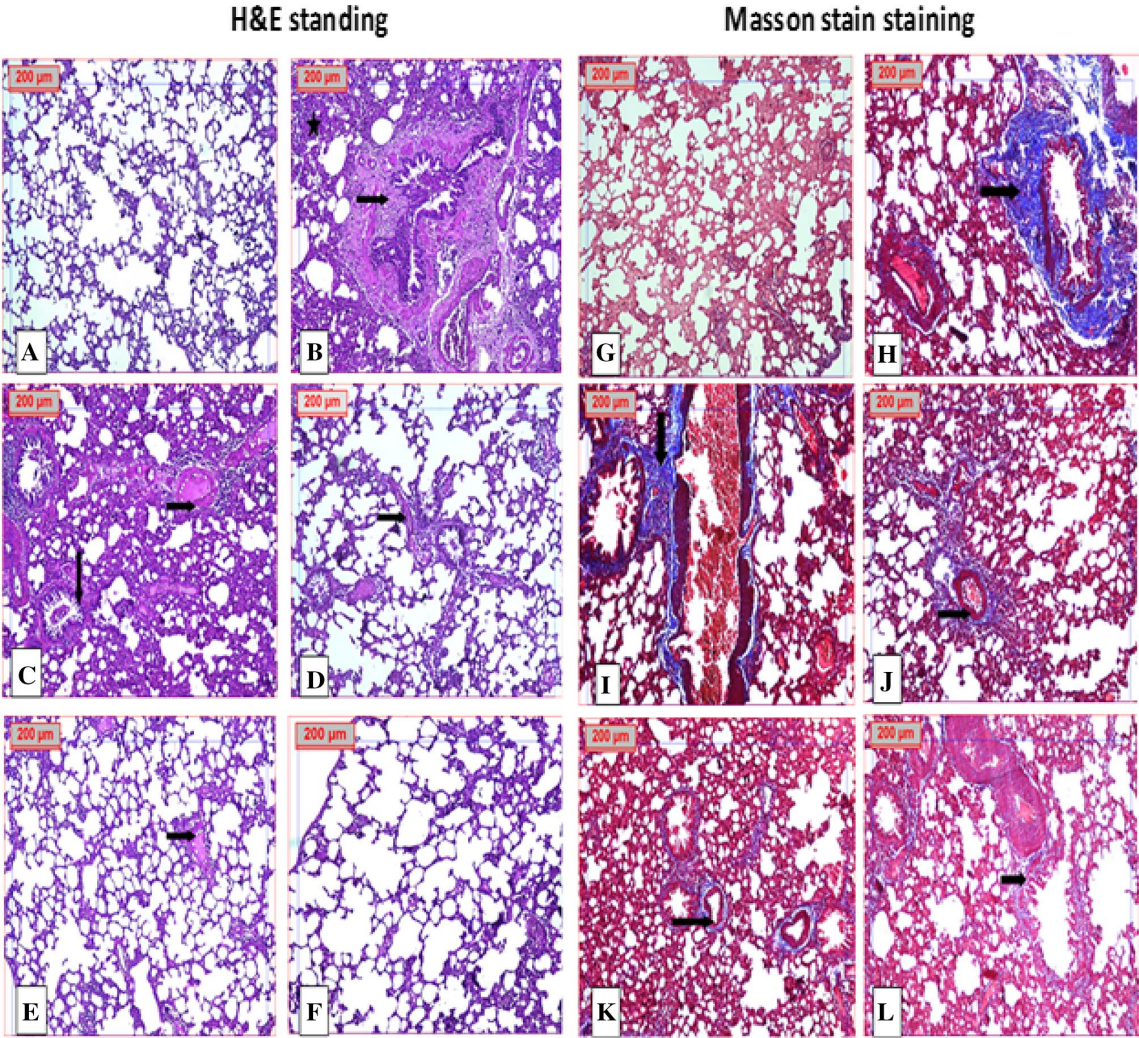
Photomicrograph of lung tissue stained H &E and Masson stains. (**A**,**G**) normal control group, (**B**,**C**,**H**,**I**) BLM treated group showed marked alveolar wall thickness, fibrosis, lymphoid aggregates and marked inflammatory cells, (**D**,**J**) Zn-NPs + BLM showed normal lung structure with minimal fibrosis deposition and minimal increase in alveolar wall thickness and (**E**,**F**,**K**,**L**) LF-Zn-NPs+ BLM revealed normal lung structure. Black arrow: fibrosis. Star: inflammation as indicated in (H&E × 100) or (Masson × 100).

As shown in (Figs. 10 and S3) H&E and confirmed by Masson stain staining of specimens showed a normal histological structure of pulmonary parenchyma with apparent intact alveolar epithelium, thin interalveolar septa and no accumulation of extracellular matrix (Fig. 10A). On the contrary, lung tissue of the BLM group was severely affected and showed diffuse pneumonia with significant thickening of the interalveolar septa due to macrophages and lymphocytic infiltration with marked congested and dilated pulmonary vasculatures and abundant inflammatory cell infiltrates (Fig. 10B,C). Groups treated with Zn-NPs showed a reduction of the thickness of the interalveolar wall with minor persistence of fibrosis (Fig. 10D). Groups treated with LF-Zn-NPs maintain a normal histological structure of the lung (Fig. 10E,F). The best result occurred with a combination of LF-Zn-NPs which showed normal lung. Additionally, the fibrosis and collagen deposit was evaluated through Masson’s Trichrome staining. In normal control group, the lung is normal with no fibrosis deposition: score 0 (Fig. 10G). BLM group showed marked destruction of lung tissue with marked fibrosis deposition at score of 4–5 (Fig. 10H,I). BML group treated with Zn-NPs showed moderate fibrosis with fibrosis score of 2 (Fig. 10J). Group treated with LF-Zn-NPs revealed normal lung structure with minimal fibrosis score of 1 (Fig. 10K,L).

## Conclusion

The current study is the first to demonstrate a rapid, one-step, cost-effective, and environmentally friendly production of Zn-NPs utilizing the ethanolic extract of *Coleus forskohlii Briq* leaves. Several approaches were used to fully characterize the biosynthesized Zn-NPs, including zeta-potential, SEM, TEM, FT-IR, and EDX. The purified LF was used to coat the biosynthesized ZnO-NPs to develop an efficient antiviral agent with immunomodulatory and anti-inflammatory properties. The biosynthesized Zn-NPs, with their small size, are supposed to accumulate more in infected cells than free compounds, increase the half-life and stability of drugs, as well as enhance the pharmacokinetic and targeting properties of the drug. The innovation in this application comes from the saturation of LF with the biosynthesized Zn-NPs to improve cellular uptake and maximize the antiviral and anti-inflammatory activities of LF. The highest efficacy of biosynthesized Zn-NPs and nanocomplex of LF-coated Zn-NPs could be a promising therapy for SARS-CoV-2 and its complications. The in vivo study shows that Zn-NPs and LF-coated Zn-NPs, act as systemic antioxidants, participating in the modulation of diverse redox mechanisms involved in physiopathological processes. Zn-NPs and LF-Zn-NPs can decrease the inflammatory response and the fibrosis score in the lung of rats treated by Bleomycin, which may lead to the development of novel strategies using Zn-NPs and LF-Zn-NPs in alleviating and/or retarding the PF development and consequences.

## Material and methods

### Plant collection and preparation of extracts

Representative samples of *C. forskohlii* Briq as traditional herbal plant was collected from the plant growing in green house in New Borg Al-Arab City, Alex, Egypt, according to guidelines and permission approved by the Research Ethical Committee at the City of Scientific research and technological applications (SRTA-City), Alexandria, Egypt. The freshly collected samples were deposited as a voucher specimens #cai.91.18.9.5 at Cairo University Herbarium (CAI) and identified and authenticated by Professor Hasnaa A. Hosni (Professor of plant taxonomy and flora) according to Boulos^73^. The plants' leaves were thoroughly cleansed in tap water before being rinsed with distilled water. They were broken up into little pieces, dried overnight at 60 °C in a hot air oven, pulverized to a particle size of 40 mesh using a mortar and pestle, and then kept at – 4 °C in an airtight container until needed. One g of a substance that had been pounded in a mortar and pestle and powdered was obtained, extracted with 15 mL of 95% ethanol, and maintained at 55 °C for a 2 h contact duration. To get a clear supernatant, each extract was centrifuged at 9000 rpm for 5 min, and the process was repeated a total of 2–3 times. The supernatant was then kept in a refrigerator at 5–10 °C until use.

### Green synthesis of Zn-NPs through *C. forskohlii* extract

The Zn-NPs applied through the current work with prepared by zinc acetate reduction using *C. forskohlii* ethanolic extract according to Melk et all approach with simple modifications^74^. The *C. forskohlii* dried extract (1 g) was dissolved in ethanol (100 mL), and added to stock of 0.5 M zinc acetate. The reaction mixture was completed to 1000 mL by ddW and boiled for 20 min at 100 °C. The reaction pH was adjusted at 12 through few drops of ammonium hydroxide. Afterward, the mixture was set at room temperature for 60 min to ensure the complete reduction of zinc acetate to Zn-NPs. The generated Zn-NPs were separated through centrifugation at 4000 rpm, washed twice in ddW, and then twice in ethanol.

### Purification of lactoferrin

Applying the protocol of Almahdy et al. with minor modifications, the raw milk is turned into skimmed milk^75^. Briefly, bovine milk was defatted by centrifuging it for 30 min at 10,000 rpm, and it was then decaseinated by bringing the pH down to roughly 4.2 using 5.0% acetic acid. The casein proteins were precipitated by centrifuging the skim milk at 4000 rpm for 20 min. The supernatant was then dialyzed for 24 h against a 50 mM Tris–HCl buffer with a pH of 7.6. According to El-Fakharany et al.'s procedures^76,77^, the acquired skim milk was used to manufacture the pure bovine LF. The resulting skim milk was put on the Mono S 5/50 GL column that had already been pre-equilibrated, and LF was eluted using a 50 mM Tris–HCl buffer with a pH of 8.0 and a NaCl gradient of 0.0 to 1.0 M. Following dialysis, the pooled LF fractions were put on a Sephacryl S100 column (5 × 150 mm, GE Health Care, Sweden), which was then eluted with 50 mM Tris–HCl buffer and 150 mM NaCl. Additionally, the resulting pooled fractions of LF were put to the affinity column of heparin-Sepharose and eluted with the same buffer that had a 0.0–1.0 M NaCl gradient. For the preparation of apo-lactoferrin (LF), about 50 mg/mL of LF had been re-dissolved in distilled H_2_O and dialyzed for 24 h against 100 mM citrate buffer, then for an additional 24 h against distilled H_2_O^78^. By using SDS-PAGE, the pure nature and molecular weight of LF were calculated. The dialyzed, lyophilized, and pooled pure LF fractions were stored at 80 °C until use. The Bradford technique was used to determine the protein concentration^79^. According to Laemmli's instructions, native polyacrylamide gel electrophoresis (PAGE) and SDS-PAGE were used to determine the molecular weight of the isolated lactoferrin^80^.

### Preparation of lactoferrin-zinc nanoparticles (LF-Zn-NPs)

A two-fold excess of Zn-NPs were added to LF, which was dissolved in 0.1 M sodium bicarbonate, to produce zinc-lactoferrin nanoparticles (LF-Zn-NPs). To remove un-ligated metal ions, thorough dialysis was then carried out against 0.1 M sodium bicarbonate. The dialyzed LF-Zn-NPs were freeze-dried to produce a solid powder after dialysis.

### Instrumental characterization of the prepared NPs

Using the Bradford and the atomic absorption methods, the quantities of LF and zinc in the formulated nano-complex were calculated^79^. The JSM 6360LA electron microscope (Tokyo, Japan), scanning electron microscopy (SEM) at 3000 and 5000X was used to assess the morphological characteristics of the produced particles. The hydrodynamic radius and zeta-potential of the prepared NPs were evaluated through Zetasizer (ZS 6.2, Malvern, Germany). Furthermore, the functional groups on the NPs surface were elucidated through Fourier transform-infrared spectroscopy (FT-IR, 8400s Shimadzu, Japan) in range of 0–4000 cm^−1^. Nanoformation of the prepared Zn-NPs and their coating with LF (LF-Zn-NPS) were confirmed through UV–VIS spectral scanning at a 200–800 nm wavelength range using spectrophotometer model UV-160A, Japan. The crystal structure and purity nature of the green synthesized Zn-NPs was estimated by X-ray diffraction (XRD) at 2θ in the range of 5° to 80° with a scan rate of 5°/min using X PERT PRO-PAN Analytical (Netherlands).

### Endotoxin levels in the prepared compound

The endotoxin levels in the prepared compounds were calculated. Using the endotoxin ELISA kit as directed by the manufacturer (Sun Long Biotech Co., Ltd.). Briefly, this ELISA kit depends on a competitive binding enzyme immunoassay approach. An antibody specific to endotoxin has been pre-coated on the microtiter plate. In the reaction, endotoxin from the sample or standard competes with a predetermined quantity of endotoxin that has been biotin-labelled for sites on a monoclonal antibody that has been pre-coated and is specific to endotoxin. The plate was washed to remove extra conjugates and unbound samples or standards. Each microplate well was then filled with and incubated with avidin conjugated to horseradish peroxidase (HRP). To each well, a TMB substrate solution was then added. A sulfuric acid solution was used to stop the enzyme–substrate reaction, and the color change was detected spectrophotometrically at a wavelength of 450 nm. A standard curve was used to determine the levels of endotoxin in the samples.

### Preparation of PBMCs cell line

According to Panda et al., the PBMCs were isolated^81^. Briefly, heparinized vials were used to collect 3 mL of peripheral blood from a single healthy participant. The Ficoll-Hispaque media solution was gently coated with the blood (they should remain as two distinct layers). Then there was instantaneous centrifugation for 30 min. at 1200 rpm, zero gradients, and 25 °C. The pale buffy coat that had developed at the interface (approximately one mL, made up of PBMCs) was aspirated right away. The aspirated layer was centrifuged twice at 2000 rpm and 25 °C for 10 min after being washed with supplement media. One mL of medium supplemented with 10% FBS was used to resuspension the produced cell pellet. Following the suggested procedure, cells were counted and diluted using complete medium to the necessary concentration.

### Determination of nanoparticles cytotoxicity

The cytotoxicity of all prepared compounds (LF, Zn-NPs, and LF-Zn-NPs) was tested in the 3-(4,5-dimethylthiazol-2-yl)-2,5-diphenyltetrazolium bromide tetrazolium reduction test (MTT assay) on normal Vero cells and PBMCs cells. 96-well sterile plates were seeded with normal cells (1 × 10^4^ cells/well) and allowed to settle overnight. Serial concentrations (0.0, 125, 250, 500, 1000 and 2000 µg/mL) of the prepared compounds were added to the cells. MTT solution (0.5 mg/mL; Sigma, USA) was added to each well after 48 h of incubation, and the plates were then incubated at 37 °C for about 5 h. Following the removal of the MTT solution, 200 µl of dimethyl sulfoxide (DMSO) was added to each well^82^. A microplate reader was then used to detect the absorbance at 570 nm (BMG LabTech, Germany). The 100% safe concentration of the preparations (EC_100_) and half maximum inhibitory concentration (IC_50_) values were calculated using the Graphpad InStat program 7. Furthermore, the effect of the prepared Zn-NPs and LF-Zn-NPs at concentrations of 250, 500 and 1000 µg/mL on the morphological changes of Vero cells were investigated by phase contrast microscope (Olympus, Japan) in comparison with LF.

### Cellular uptake and internalization of Zn-NPs and LF-Zn-NPs

#### Tagging of Zn-NPs and free LF with FITC staining method

The internalization of free LF, Zn-NPs and LF-Zn-NPs was assayed by tagging the Zn-NPs and free LF with FITC then detection in PBMCs^37^. In the beginning, a 4.8 nM stock of Zn-NPs and LF were made in deionized water (pH 7.2). About one mL of Zn-NPs and LF stock solutions were gently added to 50 µL of fluorescein isothiocyanate** (**FITC) dissolved in DMSO with constant stirring. The entire reaction was conducted in the dark and kept at 4 °C for 12 h. Following the incubation, ammonium chloride was added, bringing the total concentration to 50 mM. The mixtures were then incubated again for 2 h at 4 °C before glycerol (25% v/v) was added. The final reaction mixture was then washed with deionized water before being centrifuged at 10,000 rpm for 45 min to separate the untagged FITC.

### Cellular uptake and internalization assay

PBMCs were cultured in RPMI-1640 medium supplemented with 10% FBS for the cell internalization test in a 5% CO_2_ incubator at 37 °C. Using a six-well plate, the cells were seeded and incubated for 24 h, then 100 mg/mL of LF, Zn-NPs and LF-Zn-NPs were added at different time intervals (1, 3, 6, and 12 h). The cells were harvested, centrifuged, and washed, then suspended in PBS supplemented with 1% FBS. The internalization of free and nano compounds was examined at 488 nm using flow cytometry analysis (Partec, Germany).

### Antiviral potential of the Zn-NPs, LF and LF-Zn-NPs against SARS-CoV-2

The ability of the prepared nanocomplex and free forms of Zn and LF to block various important steps in the life cycle of SARS-CoV-2 was evaluated in vitro. The three antiviral mechanisms evaluated were binding to the ACE2 receptor, neutralizing viral recognition binding domain (RBD), Cathepsin L, and RNA-dependent RNA polymerase (RdRp).

### Blocking of the ACE2 host receptor

The BPS Bioscience #79936 ACE2/SARS-CoV-2 spike inhibitor screening kit was used to test the effect of the nanocomplex on ACE2. All tested compounds were evaluated at four different concentrations (200, 100, 50, and 25 µg/mL). A concentration–response curve for the SARS-CoV-2 spike (1–100 nM) was made to further establish a concentration-dependent increase in luminescence^83^. According to the manufacturer instructions, a 96-well nickel-coated plate was coated with an ACE2-His solution and incubated for 60 min before being cleaned. After that, the plate was incubated with a blocking buffer. The components were then combined and incubated for 60 min at room temperature while slowly shaking. 5% DMSO in water was added to the blank and positive controls. Following incubation, the SARS-CoV-2 spike (RBD)-Fc was placed into all but the blank well, and the test was left to sit while being gently shaken. After 60 min, an anti-mouse Fc-HRP was applied to the plate to produce chemiluminescence. Finally, a FluoStar Omega microplate reader was used to quantify luminescence.

### Neutralization of the SARS-CoV-2 recognition binding domain (RBD)

The BPS Bioscience #79936 ACE2/SARS-CoV-2 spike inhibitor screening kit was used to test the ability of nanocomplexes to neutralize viral spikes. A 96-well nickel-coated plate was coated with SARS-CoV-2 spike (RBD)-Fc. Then, the kit is completed as prescribed in the inserted manual. A serial concentration of the free LF, Zn-NPs, and LF-Zn-NPs (200, 100, 50, 25, and 12.5 g/mL) were added. Finally, the luminescence was quantified using a FluoStar Omega microplate reader.

### Inhibition of the RNA-dependent RNA polymerase (RdRp)

This assay evaluated the suppression of RNA synthesis in SARS-CoV-2 using the RNA-dependent RNA polymerase fluorescence kit (SARS-CoV-2 RdRp TR-FRET Assay kit). The test measures the quantity of biotinylated ATP that enters the double-stranded RNA substrate directly, which has an adverse relationship with the increase in the Time-Resolved-Förster´s Resonance Energy Transfer (TR-FRET) signals. The kit includes pure mixture of RdRp enzymes (NSP7, NSP8, and NSP12), digoxigenin-labeled RNA duplexes, and biotinylated ATP. The inhibition activity was measured by the addition of tested compounds (free LF, Zn-NPs, and LF-Zn-NPs) to the kit mixture. The TR-FRET signal is read after introducing the dye/eu-labelled acceptor and antibody to reaction.

### Protein modelling and compounds structure

Protein docking was executed utilizing Cluspro, which is accessible at the following link: https://cluspro.bu.edu/home.php. In this process, the receptor (bovine LF) structure was referenced from PDB entry 1BLF, while the ligands (SARS-CoV-2 RdRp, human ACE2, and SARS-CoV-2 RBD) structures were obtained from PDB entries 6VYO, 6M1D, and 7KJ5 respectively. The coefficient weights were calculated using the following equation: E = 0.40Erep +  − 0.40Eatt + 600Eelec + 1.00EDARS. The Cluspro platform was employed to initiate the protein docking procedure. Specifically, the receptor file used was 1BLF, and the ligand files employed were 6VYO, 6M1D, and 7KJ5. Upon completion of the computational job, the obtained results were subsequently analyzed and visualized using Chimerax software.

### In vivo evaluation of the prepared NPs activity toward Bleomycin-induced IPF

#### Experimental design

The in vivo study was carried out on 40 adult male Wistar albino rats (Animal Colony, National Research Centre, Cairo, Egypt). The adult male animals (140–180 g) were housed in appropriate plastic cages one week for acclimation. According to the NRC ethics committee (FWA 00014747), all animals were cared for by humans in accordance with the institutional standards for the handling and use of experimental animals.

### Induction of pulmonary fibrosis (IPF) and study animal groups

For IPF induction, the rats were intratracheally inoculated with 2.5 mg/kg Bleomycin hydrochloride (BLM) prepared 0.25 mL of phosphate buffered saline as described by^84^, whereas control group received similar volume (0.25 mL) of intratracheal saline. After IPF induction both normal and IPF-modeled rats were allocated arbitrarily in 4 groups (10 rats/group) included, group 1 (Control) normal rats served as control, group (2) untreated IPF-animals as positive control (2.5 mg/kg/weekly, intratracheally) for six weeks, group (3); IPF-animals that treated with Zn-NPs (50 mg/kg/day, ip) for six weeks, and group (4); IPF-animals that treated with LF-Zn-NPs (50 mg/kg/day, ip) for six weeks.

### Blood and tissue specimens

After 29 days of BLM injection, all rats were weighed and fasted overnight. Afterward, each rat was IM injected with sodium pentobarbital (9.1 mg/kg) and blood specimens were withdrawn from the retro-orbital plexus using heparinized and sterile glass capillaries. Blood sera was isolated through centrifugation (100 RCF for 10 min, undercooling), and stored at − 80 °C as aliquots for biochemical measurements. After rat’s scarification; the lung of each animal was divided equally, whereas one part was stored at − 80 °C for biochemical determinations. The other part was preserved in 10% formalin-saline buffer for histopathological processing and immunohistochemistry.

### Blood count and lung biomarkers evaluations

The total blood count was carried out through a full automated Cell blood counter (PCE-210 N, Japan) including red blood corpuscles (RBCs) count (10^6^/cm^3^), Hemoglobin (Hb) content (g/dl), hematocrit (Hct) percentage, platelets (PLT) count (10^3^/cm^3^), and total leucocytes count (TLC) count (10^3^/cm^3^). Furthermore, the lung biomarkers including C-reactive protein (CRP), ferritin, D-Dimer, Lactate dehydrogenase (LDH), fibronectin (FN), granulocyte–macrophage colony-stimulating factor (GM.CSF), and transforming growth factor beta (TGF.β) were measured using rats' reagent ELISA-kits (Sunlong Biotech Co, China).

### The Oxidative stress and pro-inflammatory cytokines determination

Using ELISA technique (Dynatech Microplate Reader Model MR 5000), the lung oxidative markers: malondialdehyde (MDA) and nitric oxide (NO) levels as well as several antioxidant enzymes including: glutathione (GSH), superoxide dismutase (SOD), catalase (CAT), and glutathione peroxidase (GPx) activity were measured using rats' reagent ELISA-kits (Sunlong Biotech Co, China). Additionally, tumor necrosis alpha (TNF-α), interlukin-1 beta (IL-1β), interlukin-4 (IL-4), interlukin-6 (IL-6), interlukin-10 (IL-10) and CD4+ levels were measured using rats' reagent ELISA-kits (Sunlong Biotech Co, China).

### Histopathology and fibrosis Type Assessment

Concerning histopathological assessment, the freshly dissected lung tissue specimens of all groups were fixed in formalin saline solution (10%) for at least 3 days. Afterward, all samples underwent a 30 min of tap water wash before being dehydrated in ascending grades of alcohol, cleaned in xylene, and fixed in paraffin. For histological analysis, serial sections of 5-μm thick were cut and stained with hematoxylin and eosin (H&E), whereas de-waxed sections of 4 m were stained with Masson's trichrome for collagen and fibrosis assessment. The fibrosis extent was assessed by Image Analysis System (Leica Qwin DW3000, Cambridge, England). The severity of IPF was expressed on a numerical scale through the fibrosis scoring system described by Ashcroft et al.^85^. Each field was assigned a score between 0 (Normal lung), 2–3 (Moderate thickening of walls without obvious damage to lung architecture), 4–5 (Increased fibrosis with definite damage to lung structure), 6–7 (Severe distortion of the structure and large fibrous areas), and 8 (Total fibrosis). The mean of the scores for all fields (5 fields/slide) was taken as the fibrotic score of the section^85^.

### Statistical analysis

The data collected was analyzed via a one-way ANOVA, then Tukey’s multiple post hoc analysis. Tests with a statistical analysis system (SAS) program software at a threshold of 0.05. The SPSS software package program (version 9) was used to apply the correlation coefficient to the provided data.

### Ethics approval and consent to participate

All methods were performed in accordance with the relevant guidelines and regulations, and the experiments done on animals are in accordance with arrive guidelines. All healthy donors of clinical blood samples provided written informed consent. All human blood samples were collected in strict accordance with and adherence to the relevant policies regarding animal handling as mandated under international, national, and/or institutional guidelines for the care of animals and were approved by the Research Ethical Committee at the City of Scientific research and technological applications (SRTA-City), Alexandria, Egypt. The experimental design was accepted by the local research ethical committee (REC) of experimental animal use at the National Research Centre, Cairo, Egypt by applying the principles of replacement, reduction and refinement (the 3Rs).

### Supplementary Information

Supplementary Information.

## Data Availability

All data generated or analyzed during this study are included in this published article.
