# Author Correction: Green synthesis, characterization, anti-SARS-CoV-2 entry, and replication of lactoferrin-coated zinc nanoparticles with halting lung fibrosis induced in adult male albino rats

**DOI:** 10.1038/s41598-023-44618-1

**Published:** 2023-10-16

**Authors:** Esmail M. El-Fakharany, Yousra A. El-Maradny, Mahmoud Ashry, Khaled G. Abdel-Wahhab, Marwa E. Shaban, Hamada El-Gendi

**Affiliations:** 1 Protein Research Department, Genetic Engineering and Biotechnology Research Institute (GEBRI), City of Scientific Research and Technological Applications (SRTA-City), New Borg El-Arab City, Alexandria, 21934 Egypt; 2 Microbiology and Immunology, Faculty of Pharmacy, Arab Academy for Science, Technology and Maritime Transport (AASTMT), Alamein, 51718 Egypt; 3 Zoology Department, Faculty of Science, Al-Azhar University, Assuit, Egypt; 4 Medical Physiology Department, National Research Centre, Giza, Egypt; 5 Pathology Department, National Research Centre, Giza, Egypt; 6 Bioprocess Development Department, Genetic Engineering and Biotechnology Research Institute, City of Scientific Research and Technological Applications (SRTA-City), New Borg El-Arab City, Alexandria, 21934 Egypt

Correction to: *Scientific Reports* https://doi.org/10.1038/s41598-023-42702-0, published online 23 September 2023

In the original version of this Article Mahmoud Ashry was incorrectly affiliated with ‘Medical Physiology Department, National Research Centre, Giza, Egypt’. Additionally, Khaled G. Abdel-Wahhab was incorrectly affiliated with ‘Pathology Department, National Research Centre, Giza, Egypt.’ Their correct affiliations are listed below.

Mahmoud Ashry:

Zoology Department, Faculty of Science, Al-Azhar University, Assuit, Egypt.

Khaled G. Abdel-Wahhab:

Medical Physiology Department, National Research Centre, Giza, Egypt.

The original Article has been corrected.

