# Supplementary material for: Green synthesis, characterization, anti-SARS-CoV-2 entry, and replication of lactoferrin-coated zinc nanoparticles with halting lung fibrosis induced in adult male albino rats
Source: Sci Rep. 2023 Sep 23;13:15921. doi: 10.1038/s41598-023-42702-0 (PMC10518009; doi:10.1038/s41598-023-42702-0)
Supplement: Supplementary file 1 — Supplementary Information. [file 41598_2023_42702_MOESM1_ESM.pdf]

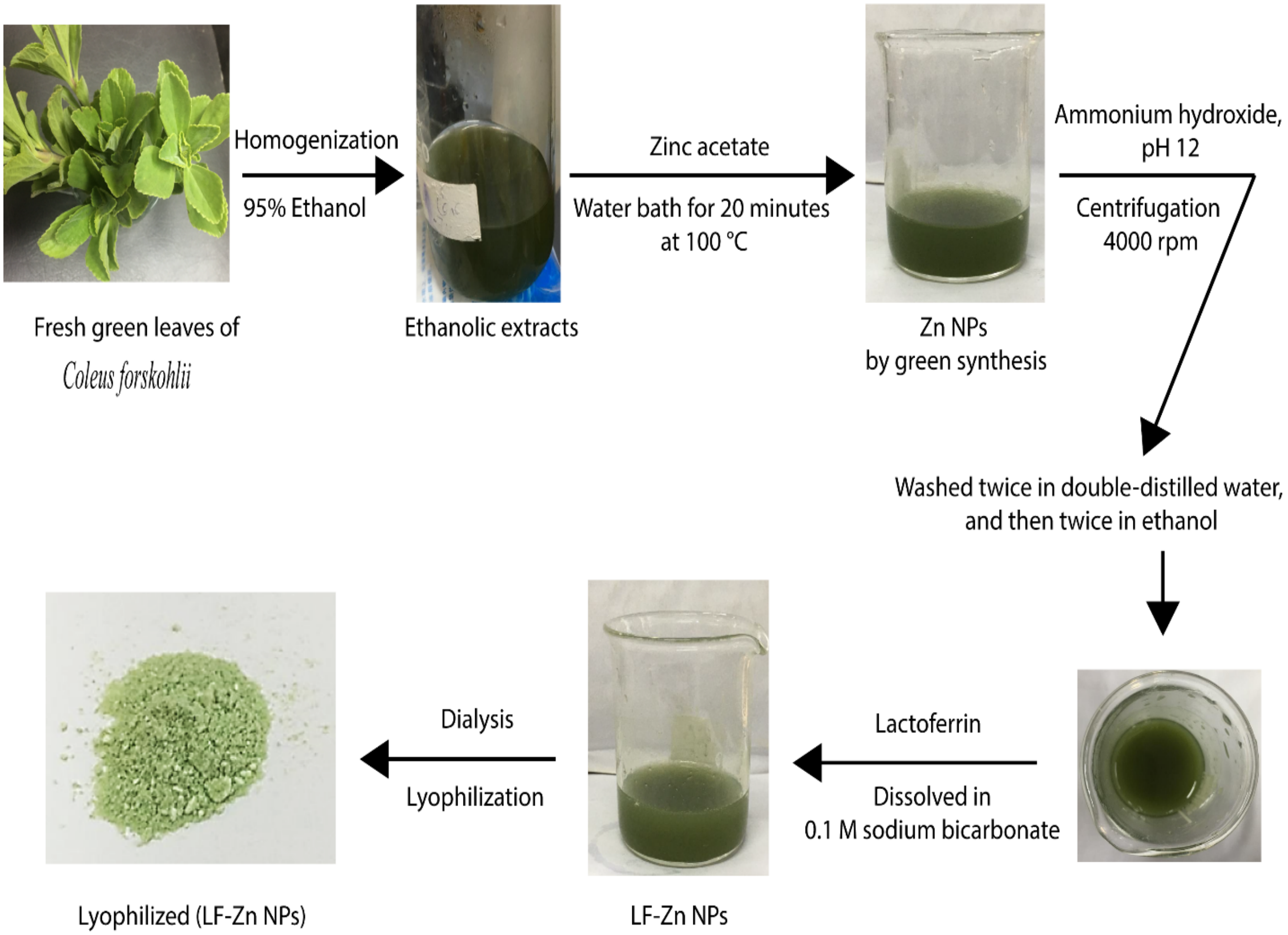

Figure S1. Schematic diagram representing the method of Zn+cLF NPs formulations form the green leaves of *Coleus forskohlii* Briq.

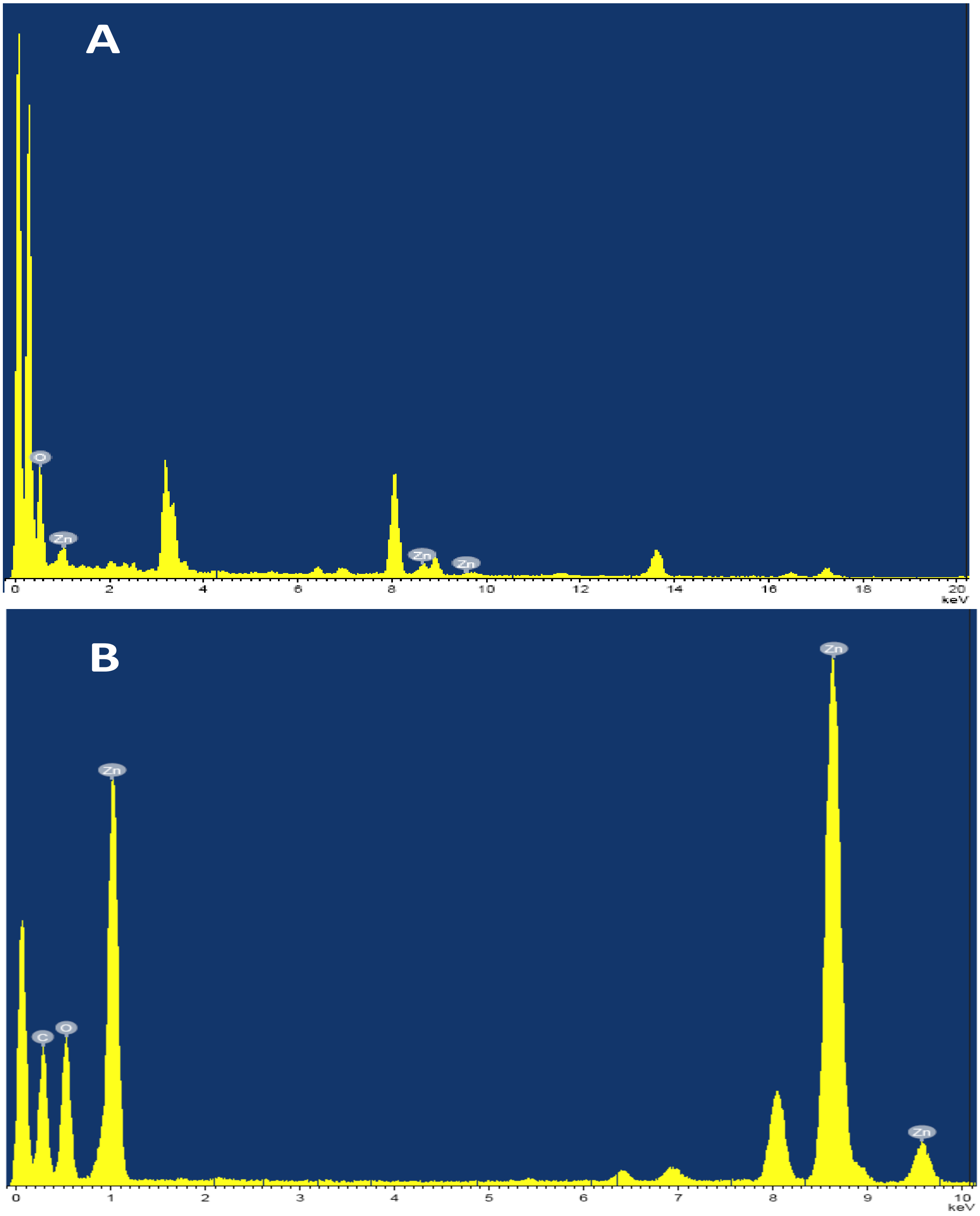

Figure S2. The EDX analysis of the prepared Zn-NPs (A) and lactoferrin coated Zn-NPs (B).

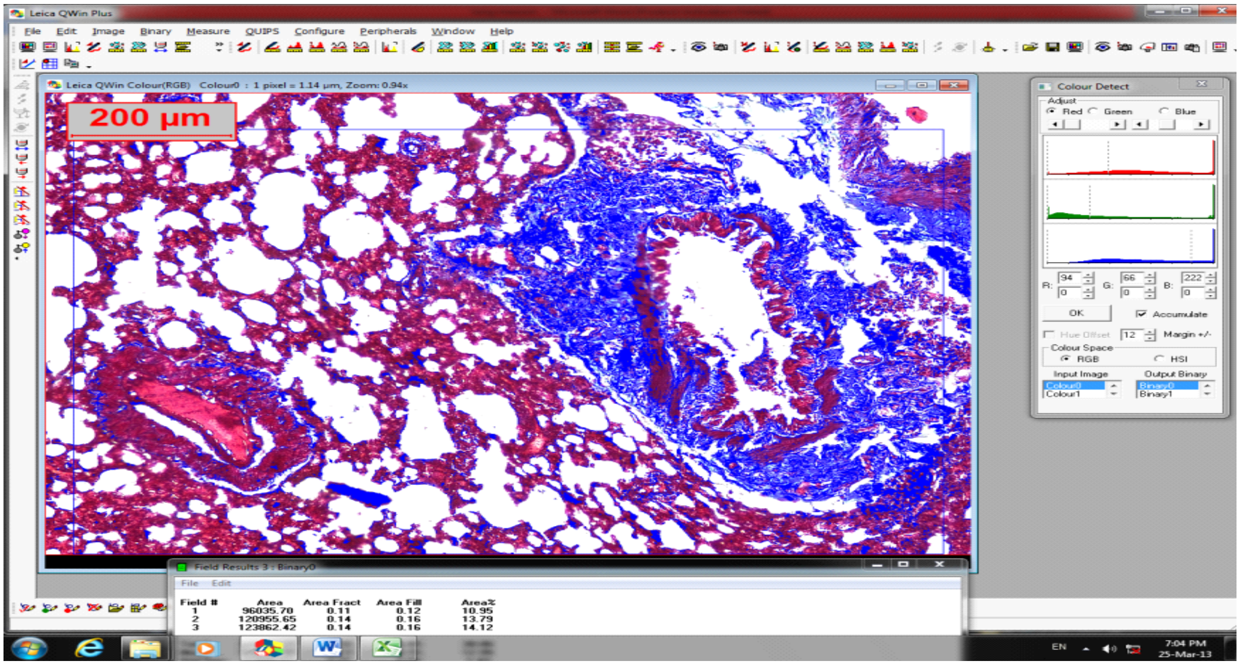

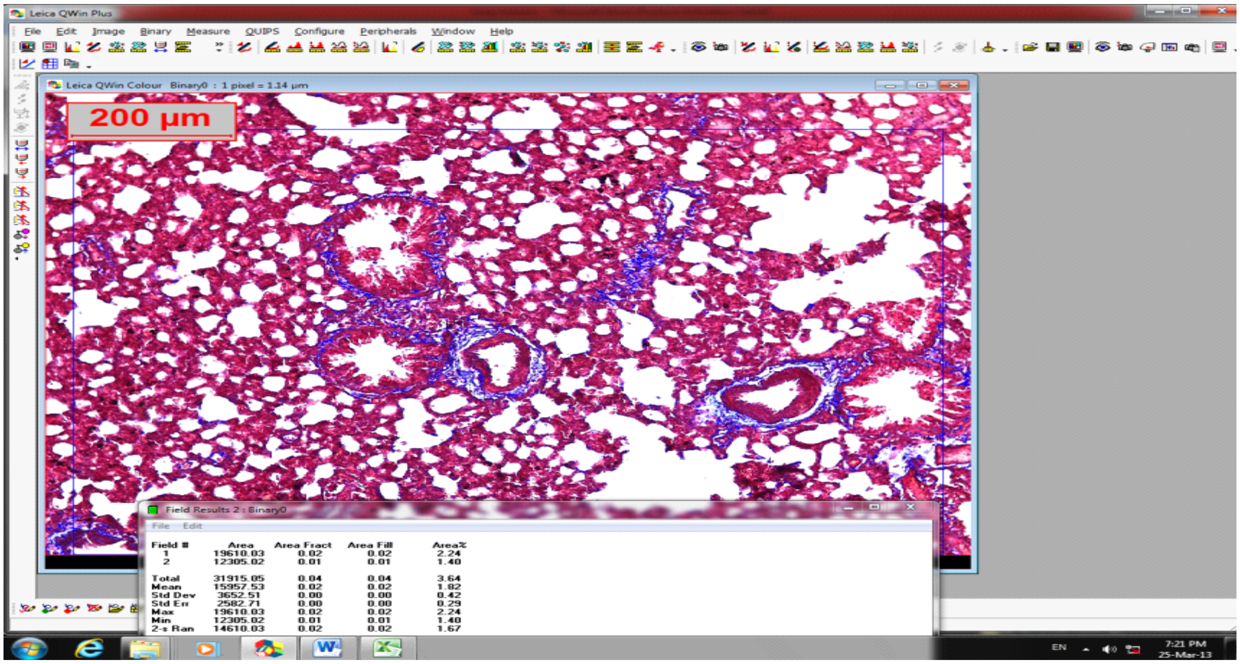

Figure S3. Photomicrograph of the lung showed area percentage fibrosis calculation using an image analysis system. Normal negative group: score 0, Positive group: score 4-5, Zn NPs+ BLM treated group: score 2, and lactoferrin coated Zn NPs+ BLM treated group score 1.

Table S1. The functional groups detected in the plant extract, Zn-NPs, and ALF-Zn-NPs

**Functional groups detected (cm^{-1})**		
**Plant extract**	**Zn-NPs**	**ALF-Zn-NPs**
3320.12	3380	3413
3001	2920	2970
2917	2850	2923
2848	1562	1564
1734	1401	1479
1639	1244	1395
1433	1021	1221
1369	838	1084
1235	675	1046
1152	612	878
1023	417	832
896		681
855		
598		
418		
